# Machine Learning-Based Classification of Pairwise Selectivity Among Human Carbonic Anhydrase I, II, IX, and XII Isoenzymes

**DOI:** 10.3390/ijms27198784

**Published:** 2026-09-30

**Authors:** Elif Gozde Hacisuleyman, Ahmet Kayraldiz, Erol Eroglu

**Affiliations:** 1 Vocational School of Health Services, Antalya Bilim University, Antalya 07190, Turkey; gozde.gokkaya@antalya.edu.tr; 2 Department of Biology, Faculty of Science, Kahramanmaras Sutcu Imam University, Kahramanmaras 46040, Turkey; akayraldiz@ksu.edu.tr; 3 Department of Mathematics and Science Teaching, Faculty of Education, Akdeniz University, Antalya 07070, Turkey

**Keywords:** carbonic anhydrase, isoenzyme selectivity, machine learning, QSAR, ChEMBL, SHAP, applicability domain, Y-randomization

## Abstract

Human carbonic anhydrase (hCA) isoenzymes participate in essential pH-regulatory processes, but conservation of their catalytic sites makes selective inhibition difficult. We developed a direct pairwise machine learning framework for selectivity among hCA I, II, IX, and XII using curated Ki data from ChEMBL. Compounds entered a binary task only when one isoenzyme was strongly inhibited (Ki ≤ 200 nM) and the paired isoenzyme was weakly inhibited (Ki > 800 nM). A provenance-conservative STRICT analysis and a BROAD sensitivity analysis were evaluated under train-only split selection, hyperparameter optimization, and a globally locked outer-test protocol. The highest observed held-out performance was obtained for CA II/CA XII (MCC = 0.748) and CA IX/CA XII (MCC = 0.718), whereas CA II/CA IX was intermediate (MCC = 0.459) and CA-I-containing tasks were generally more difficult. Because the highest point estimates arose from relatively small test cohorts, these findings should be regarded as promising and require independent external or prospective validation. All six STRICT models exceeded 100 randomized-label controls (empirical *p* = 1/101 ≈ 0.0099, the resolution floor for 100 permutations). Applicability domain and SHAP analyses indicated pair-specific signals compatible with known isoenzyme-dependent active-site differences. Overall, the framework prioritizes interpretable hCA selectivity hypotheses while explicitly defining current uncertainty and validation limits.

## 1. Introduction

Carbonic anhydrases (CAs; EC 4.2.1.1) are zinc-containing metalloenzymes that catalyze the reversible hydration of carbon dioxide to bicarbonate and protons, CO_2_ + H_2_O ⇌ HCO_3_− + H^+^. Although chemically simple, this reaction is fundamental to the rapid buffering and transport processes required to maintain acid–base homeostasis. By accelerating interconversion among CO_2_, bicarbonate, and protons, CAs support intracellular and extracellular pH regulation, respiratory CO_2_ transport, renal acid secretion and bicarbonate handling, ion transport, and metabolic fluxes that depend on bicarbonate availability [1,2]. The human α-CA family comprises 15 isoforms, 12 of which are catalytically active, and these isoforms differ in tissue distribution, cellular localization, catalytic properties, and susceptibility to inhibitors [3]. Consequently, CA activity is not a single interchangeable physiological function; rather, it is distributed among isoenzymes that participate in distinct homeostatic and disease-associated processes.

Among the isoenzymes considered in the present study, CA I and CA II are abundant cytosolic enzymes with major physiological roles, whereas CA IX and CA XII are membrane-associated isoforms that have attracted particular attention in cancer biology. CA II is an established pharmacological target in conditions such as glaucoma and has also been implicated in epilepsy, sleep-disordered breathing, metabolic disturbances, and other pH-sensitive disorders, while CA I has increasingly been investigated as a disease-associated biomarker in conditions involving erythrocyte turnover, inflammation, anemia, vascular pathology, and malignancy [4]. In contrast, CA IX and CA XII are closely linked to the adaptation of tumor cells to hypoxia and acidosis. Their extracellular catalytic domains facilitate CO_2_ hydration at the cell surface, thereby contributing to extracellular acidification while supporting maintenance of a relatively alkaline intracellular pH that favors tumor-cell survival [5,6]. Experimental perturbation of CA IX and CA XII has shown that these enzymes can support ATP preservation, cell survival, tumor growth, invasion, and metastatic behavior under acidic or hypoxic conditions [5,6]. Thus, the four isoenzymes examined here span a biologically important spectrum from broadly expressed cytosolic/homeostatic CAs to tumor-associated transmembrane CAs.

This functional diversity makes isoenzyme selectivity a central requirement in carbonic anhydrase inhibitor development. The catalytic zinc-binding machinery and the deepest portion of the active site are strongly conserved across human CAs, and many classical inhibitors, therefore, achieve high potency without sufficient isoform discrimination [3,7]. Nonselective inhibition can be undesirable when a disease-associated isoform is targeted but physiologically important isoforms, such as CA I or CA II, are inhibited at the same exposure. Conversely, in indications in which CA II itself is the desired target, excessive activity toward other isoenzymes may also complicate pharmacological interpretation. Modern medicinal chemistry strategies have, therefore, increasingly exploited amino acid differences toward the middle and entrance regions of the catalytic cavity, including the so-called “tail approach,” to direct substituents into isoform-variable subpockets while preserving a common zinc-binding pharmacophore [3,7]. From a computational perspective, the challenge is correspondingly more demanding than predicting potency against one CA in isolation: a useful selectivity model must learn the molecular features that alter relative recognition between closely related proteins rather than merely distinguish strong from weak inhibitors.

Computational efforts to model CA selectivity predate the recent expansion of machine learning. Rasti et al. used a proteochemometric framework to jointly characterize interactions between inhibitors and six mammalian isoforms (CA I, II, VII, IX, XII, and XIV), demonstrating that combined ligand–protein representations could capture determinants of isoform-dependent inhibition [8]. Pair-focused QSAR studies subsequently examined explicit selectivity endpoints. Yorulmaz et al. developed linear QSAR models for sulfonamide inhibitors of CA II and CA IX, including models based on affinity ratios and ΔpKi, and linked selectivity to interpretable molecular descriptors and docking-derived interactions involving the ligand tail and Phe131 [9]. Wang et al. extended CA II/IX selectivity analysis using CoMFA, CoMSIA, Topomer CoMFA, docking, and molecular dynamics for benzenesulfonamides [10]. Chemotype-focused DFT-QSAR studies also modeled CA II/IX selectivity for coumarins and sulfocoumarins [11] and CA II/XII selectivity for related compounds [12]. Poli et al. additionally developed a ligand-fingerprint-based cheminformatics platform for rapid structure/selectivity analysis of known CA inhibitors, illustrating how selectivity-associated motifs could be extracted without requiring target structures or explicit binding poses [13]. These studies established that CA selectivity can be represented computationally, but they were generally limited to particular chemotypes, selected isoenzyme contrasts, or specialized modeling formulations.

The availability of larger ChEMBL datasets enabled broader machine learning approaches. Tinivella et al. developed high-confidence activity classifiers for CA II, CA IX, and CA XII and inferred selectivity profiles by sequentially combining isoform-specific active/inactive predictions; notably, they used flexible bioactivity thresholds to improve class balance rather than fixing one universal threshold a priori [14]. Galati et al. took a more direct pairwise approach for CA IX versus CA II by classifying CA-IX-selective and nonselective inhibitors and then mapping predictive molecular features onto X-ray structures to rationalize selectivity [15]. More recently, Piazza et al. developed an isoform-specific ML platform for CA I, II, IX, and XII using multiple molecular representations and algorithms, followed by virtual screening, SHAP interpretation, docking, molecular dynamics, and prospective experimental testing [16]. Ghamsary et al. introduced an interpretable framework for CA II, IX, and XII that combined systematic model benchmarking with conformal prediction and counterfactual explanations, again highlighting the importance of tail region modifications for isoform differentiation [17]. Together, these studies establish a clear progression from chemotype-specific QSAR toward large-scale, interpretable, and experimentally connected ML.

Despite this progress, important methodological gaps remain. Much of the recent literature predicts activity separately for each isoform and derives selectivity from the resulting activity profile [14,16,17], whereas direct selectivity classification has been investigated primarily for selected contrasts, such as CA II versus CA IX [15]. Earlier explicit-selectivity QSAR models often represented selectivity as a continuous activity difference or ratio and were generally restricted to one chemical series or one/two isoenzyme pairs [9,10,11,12]. The literature identified for the present study did not reveal a framework that applies one prespecified binary selectivity phenotype across all six pairwise comparisons among CA I, CA II, CA IX, and CA XII while simultaneously preserving experimental provenance and evaluating the reproducibility and generalization of each pair under the same analysis architecture. This distinction is important because “selectivity” can otherwise refer to different mathematical objects across studies—separate activity calls, potency ratios, ΔpKi values, or direct binary labels—and performance values from these paradigms are not directly interchangeable.

**Figure 1:**
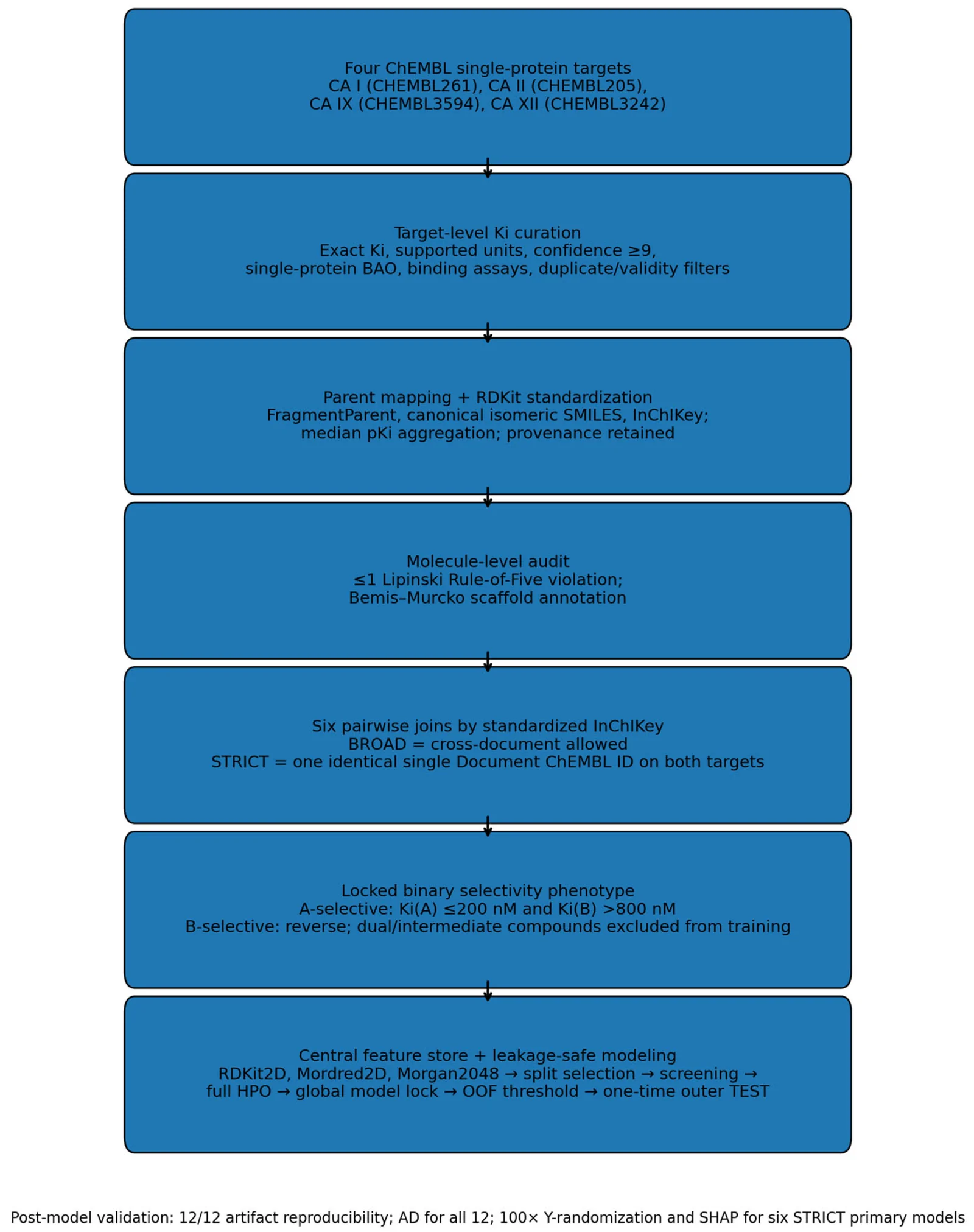
End-to-end study workflow. Four ChEMBL single-protein CA targets were subjected to exact-Ki assay curation, parent mapping and RDKit structure standardization, molecule-level median pKi aggregation, physicochemical/scaffold auditing, pairwise BROAD/STRICT matching, and a locked 200/800 nM binary selectivity phenotype. Molecular representations and all data-dependent preprocessing were handled under training-only rules, followed by split selection, screening, full HPO, a global model lock, OOF threshold selection, one-time outer-test evaluation, and post-model validation.

Accordingly, the present study was designed as a systematic pairwise selectivity analysis of CA I, CA II, CA IX, and CA XII using a single, prespecified potency-constrained phenotype. The 200/800 nM boundaries were used as operational modeling thresholds rather than as universal pharmacological or clinical cutoffs. For every ordered isoenzyme pair, a molecule was assigned to the binary modeling set only when it showed strong inhibition of one isoenzyme (Ki ≤ 200 nM) together with weak inhibition of the paired isoenzyme (Ki > 800 nM). Dual-active, dual-weak, and intermediate/mixed molecules were excluded from classifier training but retained for audit. This produced six direct classification tasks—CA I/II, CA I/IX, CA I/XII, CA II/IX, CA II/XII, and CA IX/XII—with a fixed label orientation and identical biological thresholds. To address uncertainty in experimental provenance, a conservative STRICT analysis required the two target measurements contributing to a pairwise molecule to originate from the same single ChEMBL document, whereas a BROAD analysis served as an end-to-end sensitivity analysis without the shared-document requirement. The modeling workflow then compared multiple 2D molecular representations and classifier families under train-only split selection and hyperparameter optimization, locked all final model decisions before outer-test evaluation, and complemented predictive performance with bootstrap confidence intervals, applicability domain analysis, 100-fold Y-randomization, independent artifact reproducibility, and SHAP-based molecular interpretation. The primary rule imposes at least a fourfold Ki separation between the preferred and paired isoenzymes. Medicinal chemistry studies of CA inhibitors commonly report selectivity as a continuous selectivity index rather than a universal cutoff, with examples spanning modest to very large fold differences [18,19]. By comparing cytosolic–cytosolic, cytosolic–tumor-associated, and tumor-associated–tumor-associated pairs within one frozen framework, the study was designed not only to identify which CA selectivity contrasts show higher predictive separability under the present framework, but also to determine whether the molecular features associated with selectivity are shared or pair specific. The complete analysis architecture is summarized in Figure 1.

## 2. Results

After upstream structure curation and the maximum-one-Lipinski-violation filter, the target-level molecule tables entering pairwise discovery contained 5117 unique molecules for CA I, 5524 for CA II, 4928 for CA IX, and 4019 for CA XII. Pairwise matching and application of the locked selectivity phenotype yielded BROAD binary datasets ranging from 161 to 932 molecules and STRICT datasets ranging from 152 to 905 molecules. All 12 final datasets passed integrity checks, with row counts equal to unique standardized InChIKey counts and no standardized-SMILES representation mismatches. Detailed target-level and pairwise attrition is available in Supplementary Tables S1–S3.

The STRICT provenance criterion retained 86.2–97.1% of the corresponding BROAD binary molecules (Table 1), indicating that same-document filtering reduced sample size only modestly for most pairs. Class composition, however, differed markedly among tasks. CA-I-containing tasks were particularly imbalanced: CA I vs. CA II contained only 23 CA-I-selective molecules versus 775 CA-II-selective molecules in STRICT, while CA I vs. CA XII contained 37 versus 724. By contrast, CA II vs. CA IX (176 vs. 124) and CA IX vs. CA XII (52 vs. 100) were substantially more balanced. The union of all final pairwise datasets comprised 1572 unique standardized molecules in the central feature store.

Because the 200/800 nM definition is operational, we performed a post hoc threshold-sensitivity analysis without changing or re-selecting the primary models. More stringent definitions (100/800, 200/1000, and 100/1000 nM) progressively reduced cohort size, especially the minority class, whereas a more permissive 300/600 nM definition increased cohort size but reduced the minimum potency separation to twofold (Supplementary Table S14; Supplementary Figure S7). When the originally locked primary TEST predictions were examined only within nested stricter subsets, the qualitative ordering was broadly retained for CA II/CA XII, CA IX/CA XII, and CA II/CA IX, whereas CA-I-containing results remained more variable and strongly dependent on the few available minority examples (Supplementary Table S15). These post hoc subset analyses were descriptive only—no retraining, repartitioning, HPO, or threshold retuning was performed.

TRAIN-only split diagnostics selected scaffold-disjoint partitions for 7 of the 12 STRICT/BROAD datasets and stratified random partitions for the remaining 5—the maxi–min/KS-like candidate was not selected for any dataset. Among the six primary STRICT tasks, three used scaffold-disjoint outer splits (CA I vs. CA IX, CA I vs. CA XII, and CA II vs. CA IX) and three used stratified random outer splits (CA I vs. CA II, CA II vs. CA XII, and CA IX vs. CA XII). The TRAIN-only selector scores for all three candidate outer splits, together with the selected active split, are reported for all 12 datasets in Supplementary Table S4.

No single molecular representation or classifier family was uniformly optimal across the six primary tasks. Final STRICT winners included logistic regression for three pairs, ExtraTrees for CA I vs. CA II, random forest for CA II vs. CA XII, and RBF-SVC for CA IX vs. CA XII. Winning feature spaces ranged from RDKit2D or Mordred2D alone to Morgan2048 and combined fingerprint/descriptor representations (Table 2). All split, feature, model, hyperparameter, and decision threshold choices were completed from outer-training data before the corresponding final test set was evaluated. Full-HPO winners for all 12 STRICT/BROAD datasets are given in Supplementary Table S5.

The final OOF-selected operating thresholds differed from the model-default cutoff for five of the six primary models (Supplementary Table S16). OOF threshold tuning increased MCC most noticeably for CA I/CA II (0.436 to 0.527) and more modestly for the other affected models. CA I/CA XII retained the default 0.5 cutoff. For the RBF-SVC CA IX/CA XII model, the default decision function cutoff was 0 rather than 0.5, and the selected OOF cutoff was 0.0133. These thresholds were fixed before final TEST evaluation.

**Figure 2:**
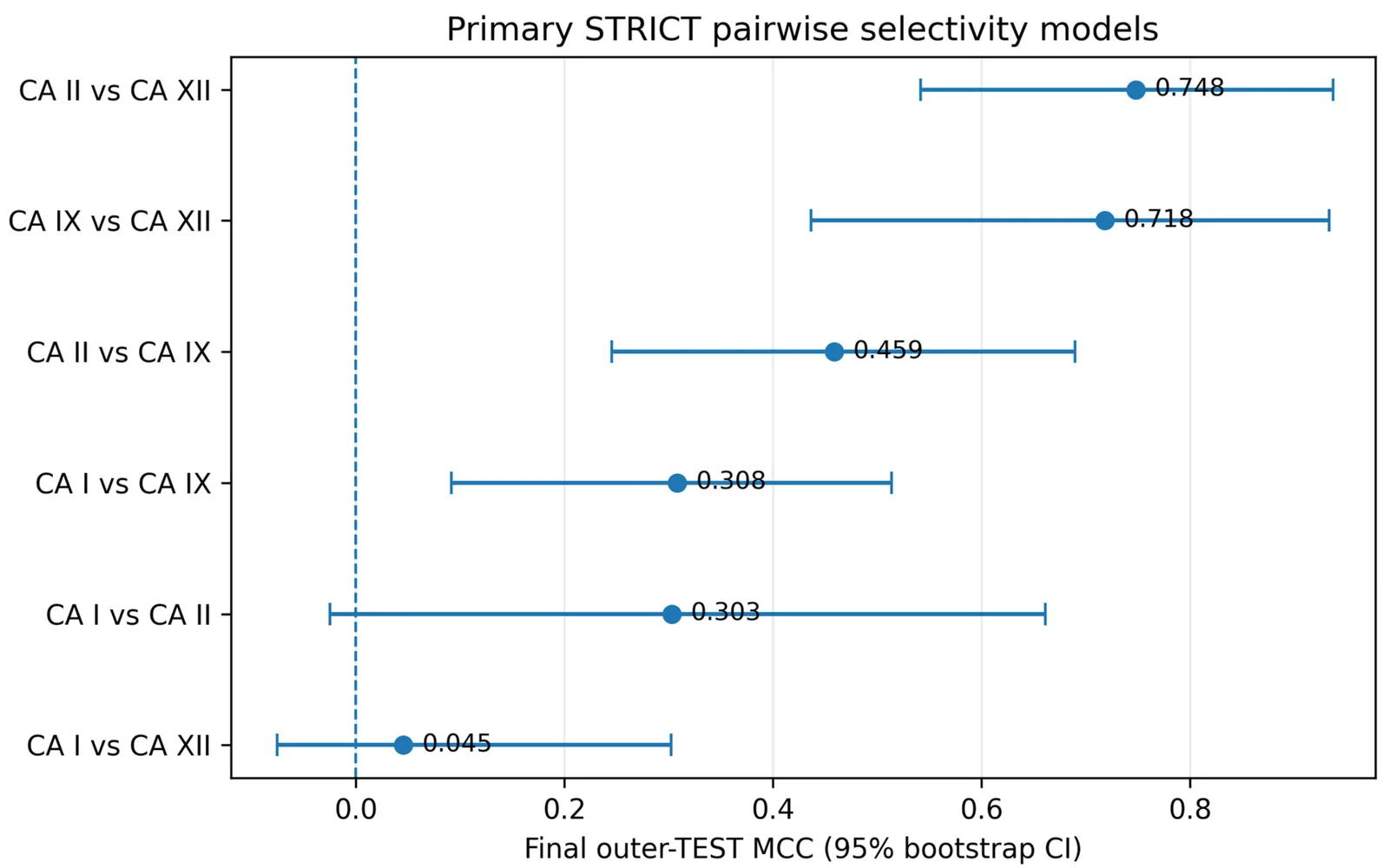
Final outer-test MCC for the six primary STRICT models with 95% bootstrap confidence intervals (1000 stratified resamples). The dashed vertical line marks MCC = 0. Point estimates are shown next to the markers. The figure highlights substantial heterogeneity in generalization across isoenzyme pairs and the wide uncertainty associated with some class-imbalanced tasks.

Final outer-test performance differed markedly across the six pairwise tasks (Table 2; Figure 2). Among the six held-out evaluations, the highest observed point estimate was obtained for CA II vs. CA XII, with MCC = 0.748 (95% CI 0.541–0.937), macro-PR-AUC = 0.991, balanced accuracy = 0.818, and macro-F1 = 0.857. CA IX vs. CA XII also showed a high observed point estimate, with MCC = 0.718 (95% CI 0.436–0.933), macro-PR-AUC = 0.933, balanced accuracy = 0.859, macro-F1 = 0.859, and minority recall = 0.818. The complete 12-model locked-test summary is provided in Supplementary Table S6.

CA II vs. CA IX showed intermediate but clearly positive performance, with MCC = 0.459 (95% CI 0.245–0.690), macro-PR-AUC = 0.768, balanced accuracy = 0.731, and minority recall = 0.720. CA I vs. CA IX produced a more modest MCC of 0.308, but its bootstrap interval remained above zero (95% CI 0.092–0.514). In contrast, CA I vs. CA II yielded MCC = 0.303 with a wide interval spanning zero (−0.025–0.661) and minority recall of 0.200. CA I vs. CA XII was the weakest task, with MCC = 0.045 (95% CI −0.075–0.302), macro-PR-AUC = 0.515, balanced accuracy = 0.530, and minority recall = 0.143, indicating little useful outer-test discrimination under the primary STRICT definition.

The confidence intervals were necessarily wide for the smallest locked outer-test sets, particularly CA IX vs. CA XII (n = 31) and CA II vs. CA XII (n = 40). Although their lower bootstrap limits remained above zero, the small numbers of independent TEST compounds impose substantial sampling uncertainty, and bootstrap resampling cannot create information that is absent from a small cohort. Accordingly, these values should be interpreted as the highest observed performance within the present held-out evaluation, not as definitive estimates of generalizable performance or of a general molecular mechanism.

**Figure 3:**
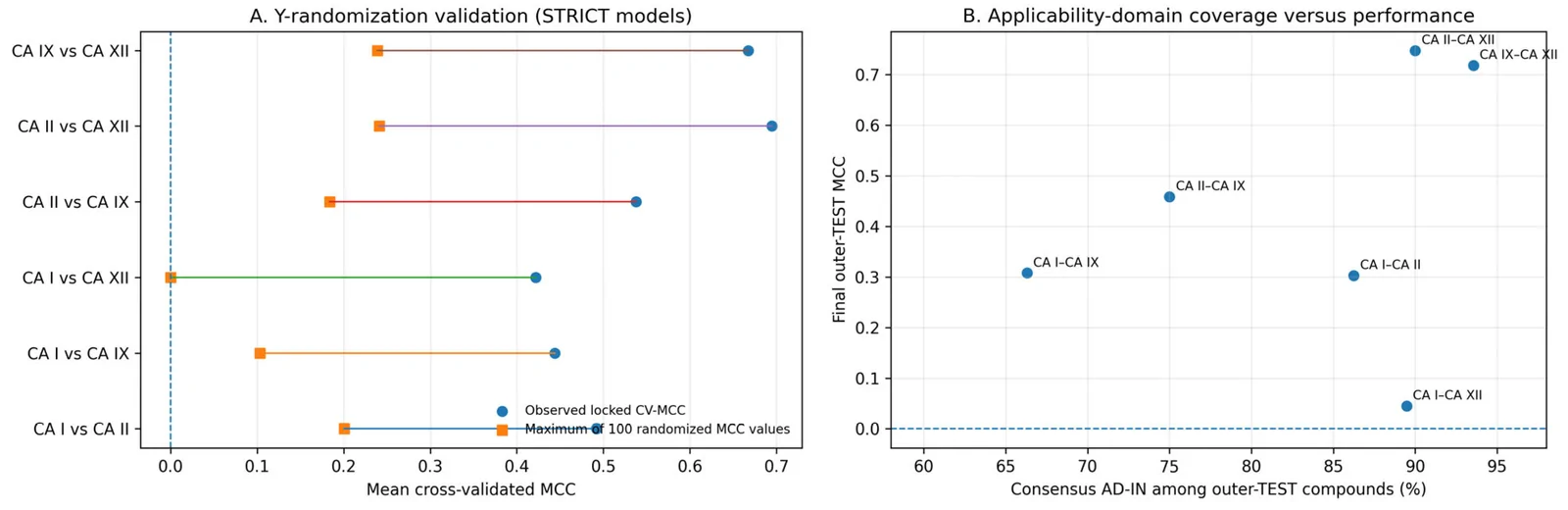
Validation of the six primary STRICT models. (**A**) Observed locked cross-validated MCC compared with the maximum MCC among 100 Y-randomization permutations for each task. All six empirical *p*-values were 1/101 = 0.00990. (**B**) Consensus AD coverage among locked outer-test compounds plotted against final outer-test MCC. Consensus AD-IN required both Morgan–Tanimoto and PCA-leverage criteria. Y-randomization addresses chance correlation in model development and should not be interpreted as a substitute for outer-test generalization.

For every primary STRICT model, the observed locked cross-validated MCC exceeded the maximum value obtained in 100 randomized-label experiments (Figure 3A). The empirical exceedance value was 1/101 ≈ 0.00990 for all 6 tasks, which is the minimum resolution attainable with 100 permutations. The maximum randomized MCC ranged from 0.000 for CA I vs. CA XII to 0.241 for CA II vs. CA XII, whereas observed locked CV-MCC values ranged from 0.422 to 0.695. The observed comparator was the CV-MCC of the HPO-selected final configuration, while the randomized-label runs retained that configuration without repeating HPO or threshold optimization; accordingly, this analysis is a fixed-configuration chance-correlation stress test rather than a fully exchangeable permutation test of the complete model selection pipeline. For CA I vs. CA XII, all 100 randomized runs yielded MCC = 0.000 and macro-PR-AUC = 0.500. Inspection of the frozen configuration (L1-regularized logistic regression, C = 0.005) and checkpoint outputs indicates a degenerate non-discriminative random label regime consistent with constant-score/single-class predictions—this pattern was not interpreted as stronger biological evidence than for the other pairs. Thus, Y-randomization was used only as a chance-correlation check and not as evidence of outer-test generalizability. See Supplementary Table S9 and Supplementary Figure S2 for the complete 100-permutation comparison.

Consensus AD coverage among STRICT outer-test compounds ranged from 66.3% to 93.5% (Figure 3B). CA IX vs. CA XII showed the highest coverage (29/31, 93.5%), followed by CA II vs. CA XII (36/40, 90.0%) and CA I vs. CA XII (136/152, 89.5%). The latter pair is notable because its outer-test MCC was only 0.045 despite high-consensus AD coverage, showing that poor performance could not simply be attributed to most test compounds lying outside the defined chemical domain. CA I vs. CA IX had the lowest-consensus AD coverage (120/181, 66.3%), so chemical-space extrapolation may have contributed to its more modest generalization, although the present analysis does not establish causality. AD thresholds, component counts, explained variance, and STRICT/BROAD coverage are reported in Supplementary Table S8 and Supplementary Figure S3.

An additional subgroup analysis indicated, within the present dataset, that AD status should not be equated with label reliability (Supplementary Table S18; Supplementary Figure S8). For CA I/CA XII, the AD-IN subset contained 136/152 TEST molecules yet remained non-discriminative (MCC = −0.046; balanced accuracy = 0.473), whereas CA I/CA IX performed substantially better in AD-IN than AD-OUT molecules (MCC 0.520 vs. 0.066). For CA II/CA XII and CA IX/CA XII, the AD-OUT subsets contained only four and two compounds, respectively, so their subgroup metrics were too unstable for substantive interpretation. Within the present dataset, these findings support treating consensus AD as a chemical-space similarity/leverage diagnostic rather than as a measure of selectivity-label correctness or experimental reliability.

Sensitivity analyses using the BROAD provenance definition did not produce a uniform directional change in performance. Relative to STRICT, outer-test MCC decreased from 0.459 to 0.396 for CA II vs. CA IX, from 0.748 to −0.031 for CA II vs. CA XII, and from 0.718 to 0.614 for CA IX vs. CA XII. Conversely, MCC increased from 0.303 to 0.402 for CA I vs. CA II, from 0.308 to 0.518 for CA I vs. CA IX, and from 0.045 to 0.289 for CA I vs. CA XII. The full end-to-end STRICT/BROAD sensitivity matrix is reported in Supplementary Table S7 and Supplementary Figure S1.

These STRICT–BROAD differences were treated as descriptive robustness results rather than causal estimates of document provenance. The optimization pipeline was run independently for each variant, so the selected feature representation, classifier family, and in some cases the active outer split differed between STRICT and BROAD. The CA II vs. CA XII comparison was the most extreme example: the STRICT winner used a random outer split with Morgan2048 and random forest, whereas the BROAD winner used a scaffold-disjoint split with Morgan2048 and logistic regression. Accordingly, the large MCC difference for this pair cannot be attributed solely to the same-document provenance filter.

Residual activity-level heterogeneity was also audited within the frozen STRICT datasets (Supplementary Table S19). More than 99% of target-side molecule records were represented by a single retained measurement and a single assay in the final molecule-level tables, and no high-variability flag was present. Nevertheless, the same-document STRICT rule cannot harmonize unrecorded or incompletely standardized experimental differences, such as buffer composition, pH, temperature, protein construct, substrate concentration, assay implementation, or laboratory practice—these factors remain a potential source of Ki and label variation.

SHAP analysis indicated that the molecular representations driving classification were pair-specific rather than dominated by a single universal feature. The leading global feature was Morgan bit 1452 for CA II vs. CA XII, Morgan bit 1440 for CA IX vs. CA XII, SMR_VSA4 for CA II vs. CA IX, Morgan bit 935 for CA I vs. CA II, the RDKit bicyclic-fragment count (fr_bicyclic) for CA I vs. CA IX, and the Mordred descriptor BCUTd-1h for CA I vs. CA XII. Primary model SHAP methods and top features are summarized in Supplementary Tables S10 and S11.

**Figure 4:**
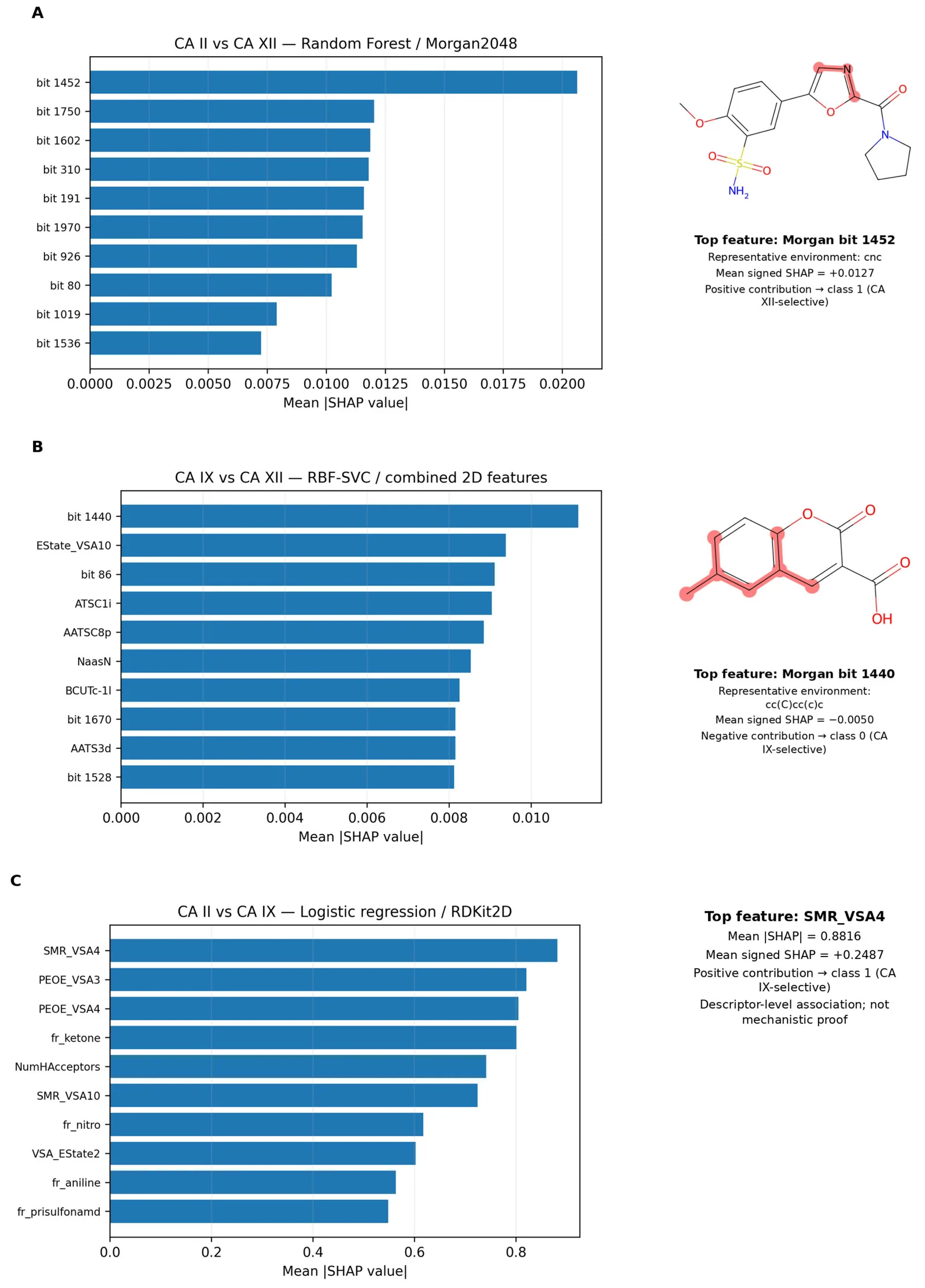
Representative global SHAP profiles and structural feature mappings for three primary STRICT models. (**A**) CA II vs. CA XII random forest/Morgan2048: the ten highest-importance Morgan bits and a representative molecular environment for top-ranked bit 1452. (**B**) CA IX vs. CA XII RBF-SVC/combined 2D features: top-ranked features and the representative environment for Morgan bit 1440. (**C**) CA II vs. CA IX logistic regression/RDKit2D: top descriptor features, led by SMR_VSA4. Bar lengths represent mean absolute SHAP values. Signed SHAP directions shown in the annotation cards refer to the canonical class orientation (class 0 = first isoenzyme selective; class 1 = second isoenzyme selective). These associations should not be interpreted as direct mechanistic proof of protein–ligand interactions.

Three representative models are shown in Figure 4. For CA II vs. CA XII, Morgan bit 1452 was the highest-ranked feature (mean |SHAP| = 0.0206) and mapped in a representative training molecule to a radius-1 “cnc” environment—its mean signed SHAP was positive (+0.0127), corresponding to contribution toward class 1 (CA-XII-selective). For CA IX vs. CA XII, Morgan bit 1440 was the leading feature (mean |SHAP| = 0.0112) and mapped to an aromatic radius-2 environment represented as “cc(C)cc(c)c”—its mean signed SHAP was negative (−0.0050), corresponding to contribution toward class 0 (CA-IX-selective). For CA II vs. CA IX, the leading descriptor SMR_VSA4 had mean |SHAP| = 0.8816 and positive mean signed SHAP (+0.2487), corresponding to contribution toward class 1 (CA-IX-selective). Complementary global SHAP profiles for CA I vs. CA II, CA I vs. CA IX, and CA I vs. CA XII are shown in Supplementary Figures S4–S6.

These findings identify model-associated structural or physicochemical correlates of pairwise selectivity. They are not, by themselves, evidence that the highlighted fragments or descriptors directly contact isoenzyme-specific active-site residues. Structural interpretation against experimentally characterized CA I/II/IX/XII binding pockets is, therefore, reserved for the Discussion Section.

All 12 final STRICT/BROAD models passed the independent artifact-reproducibility audit. After reloading the saved estimators and their saved preprocessed test matrices, prediction scores were reproduced to numerical differences on the order of 10^−16^, and the stored OOF-derived thresholds, final class labels, and recorded performance metrics were recovered exactly within numerical tolerance. The reproducibility check was completed before AD, Y-randomization, and SHAP outputs were used for manuscript interpretation. The complete reproducibility audit is provided in Supplementary Table S12.

## 3. Discussion

The present study shows that pairwise carbonic anhydrase selectivity produced markedly different levels of predictive separability under the present datasets and modeling conditions. When the same potency-constrained phenotype was applied across all six comparisons, the highest held-out MCC values were observed for CA II vs. CA XII and CA IX vs. CA XII, CA II vs. CA IX showed intermediate discrimination, and the CA-I-containing tasks were generally weaker. These differences should not be interpreted as proof that some isoenzyme pairs are intrinsically or biologically more “learnable” than others. The tasks differ simultaneously in total sample size, minority-class support, chemotype composition, experimental provenance, selected split, molecular representation, classifier family, and genuine structural biology. The present results, therefore, quantify pair-specific predictive separability within this particular data-generating and validation framework rather than an intrinsic ranking of isoenzyme distinguishability.

Between-pair differences in held-out performance also reflect the stringency of the selected outer split. The two highest-scoring STRICT tasks, CA II/CA XII and CA IX/CA XII, used stratified random outer splits and showed mean TEST-to-TRAIN maximum Tanimoto similarities of 0.674 and 0.618, respectively, whereas the scaffold-disjoint CA II/CA IX task showed a lower value of 0.471 (Supplementary Table S8). Random splitting, therefore, provided less stringent chemical-space separation for these two tasks than scaffold-disjoint evaluation. However, split type alone does not explain the performance ordering: CA I/CA II also used a random split and had an even higher mean TEST-to-TRAIN similarity (0.700), yet its locked TEST MCC was only 0.303. Accordingly, direct between-pair comparisons should be interpreted as reflecting the joint effects of split stringency, sample size and class balance, chemotype composition, molecular representation/model choice, and the underlying structure-selectivity signal rather than split type or biology alone.

Earlier computational studies approached CA selectivity from several complementary directions. Proteochemometric modeling combined ligand and isoenzyme information across multiple CA targets [8], while chemotype-focused QSAR studies modeled CA II/IX or CA II/XII selectivity using activity differences or selectivity indices [9,11,12]. More recent machine learning studies have moved toward large-scale ligand classification. Tinivella et al. trained separate activity classifiers for CA II, IX, and XII and inferred selectivity by sequentially combining the predicted active/inactive labels [14]. Galati et al. directly classified CA-IX-selective versus nonselective inhibitors relative to CA II and then mapped important ECFP features onto crystallographic structures [15]. Piazza et al. subsequently developed isoform-specific activity classifiers for CA I, II, IX, and XII and combined SHAP analysis with docking, molecular dynamics, and prospective experimental testing [16]. Most recently, Ghamsary et al. reported interpretable activity classifiers for CA II, IX, and XII with conformal prediction and counterfactual explanations, again emphasizing the importance of tail region changes for isoform differentiation [17]. Against this background, the present design differs in that each task directly asks for the direction of pairwise selectivity among compounds that already satisfy a high-confidence target-active/off-target-weak phenotype and applies the same 200/800 nM rules to all six possible pairs. The resulting models, therefore, do not replace isoform-specific activity models; rather, they address a narrower question: given a molecule that meets the prespecified high-confidence selectivity phenotype for a pair, which member of that pair is preferentially inhibited?

The structural basis of CA inhibitor recognition provides a useful framework for interpreting why pairwise ligand selectivity signals differ. Representative experimental structures include inhibitor-bound CA I (PDB 1AZM) [20], the refined CA II structure (PDB 1CA2) [21], the catalytic domain of CA IX complexed with acetazolamide (PDB 3IAI) [22], and the extracellular catalytic domain of CA XII complexed with acetazolamide (PDB 1JD0) [23]. These structures share the canonical alpha-CA catalytic architecture. In CA II numbering, the catalytic Zn^2+^ ion is coordinated by His94, His96, and His119 together with a water/hydroxide ligand, Thr199 and the ordered solvent network participate in catalysis, and His64 serves as the principal proton-shuttle residue. Consequently, the deepest region of the active site is highly conserved, which explains why a zinc-binding group by itself often provides potency but limited isoform selectivity. Isoform discrimination is more readily generated toward the middle and entrance regions of the cavity, where amino acid substitutions alter steric volume, polarity, water organization, and the direction available to inhibitor tails [3,5,24,25].

A useful structural concept is the “selective pocket” or selective drug targetability region toward the active-site exit [24]. Using CA II numbering, several residues within approximately 5–15 Å of the catalytic zinc differ systematically among CA I, II, IX, and XII (Table 3) [5]. Notably, CA I contains bulky His67, Phe91, and Tyr204, CA II contains Phe131, CA IX contains the smaller Val131 together with a relatively hydrophobic Leu91/Leu135 environment, and CA XII combines Lys67, Thr91, Ala131, Ser135, and Asn204, producing a markedly different distribution of size and polarity. A recent structure-annotated analysis of 130 sulfonamide cocrystal structures identified the 130s subpocket and entrance-zone contacts as recurrent sources of CA II/IX/XII selectivity [25]. Accordingly, the mechanistic interpretation of the present SHAP results is focused on whether model-associated molecular features are chemically compatible with these known cavity differences, not on claiming that a SHAP feature establishes a specific protein–ligand contact.

CA II vs. CA XII showed the highest observed performance in the present locked outer-test evaluation (MCC = 0.748; macro-PR-AUC = 0.991), with a bootstrap MCC interval that remained well above zero. The final model used Morgan2048 features and random forest, suggesting that local substructural environments carried useful discriminatory signals in the present dataset. Given the 40-compound TEST cohort, this observation remains exploratory and requires independent validation. The leading SHAP feature was Morgan bit 1452. Its representative mapped environment contained a small nitrogen-containing heteroaromatic ‘cnc’ motif and its mean signed SHAP value was positive, i.e., it was associated with the CA-XII-selective direction in the present label convention. Other highly ranked mapped bits included aromatic, phenolic, amino-containing, and methoxy environments (see Supplementary Table S11). Because these are hashed fingerprint features and because a bit may represent more than one atom environment, the individual fragments should be viewed as structural correlates rather than unique pharmacophores.

Nevertheless, the direction of the signal is chemically compatible with known CA II/CA XII structural differences. Relative to CA II, CA XII replaces Ile91 with Thr, Phe131 with Ala, Val135 with Ser, and Leu204 with Asn, while Asn67 is replaced by Lys [5]. This combination simultaneously reduces steric bulk at position 131 and increases the polar character of several residues surrounding the selective region. The CA XII crystal structure itself identified the nearby 130s segment as a region in which the active-site composition differs from other isoforms [23]. The large-scale crystallographic review of Ahmad et al. likewise highlighted the Phe131/Gly132/Val135 pattern in CA II versus Ala131/Ser132/Ser135 in CA XII as an exploitable structural difference [25]. Thus, heteroaromatic nitrogen atoms and appropriately positioned hydrogen-bond acceptors may plausibly be more readily accommodated in a polar, less sterically constrained CA XII environment, whereas bulky or differently oriented groups may be disfavored by Phe131 in CA II. This interpretation is a structural hypothesis consistent with the SHAP pattern—direct assignment of bit 1452 to a specific CA XII residue would require matched protein–ligand structures, docking constrained by experimental structures, or prospective crystallography.

The CA IX vs. CA XII task also showed a high observed held-out score (MCC = 0.718; macro-PR-AUC = 0.933). The leading Morgan feature, bit 1440, mapped to a representative methyl-substituted aromatic environment and had a negative mean signed SHAP value, corresponding to the CA-IX-selective direction. Several additional high-ranking negative features represented fluorinated or substituted aromatic environments, whereas positive features among the leading mapped bits included more polar motifs, such as an aliphatic amine-containing environment, a hydroxyalkyl/heteroatom chain, and a sulfone-containing environment (see Supplementary Table S11). This is notable because CA IX and CA XII differ in the hydrophobic character of the same selectivity region: CA IX contains Leu91, Val131, and Leu135, whereas CA XII contains Thr91, Ala131, and Ser135 [5]. Singh et al. specifically noted that the CA IX active site is more hydrophobic than that of CA XII and proposed that hydrophobic ligand groups can exploit this difference [5]. The 31-compound TEST set and its wide bootstrap interval nevertheless limit precision, and these results should, therefore, be considered exploratory evidence rather than definitive proof of broad generalizability.

The present SHAP direction, therefore, supports a plausible model in which hydrophobic aromatic substitution favors CA IX more often than CA XII within the high-confidence phenotype, while selected polar side-chain environments can shift predictions toward CA XII. The interpretation should not be reduced to a simple hydrophobicity rule, because CA IX also contains the positively charged Arg60 and Gln67, whereas CA XII contains Thr60 and Lys67, and ligand orientation and water-mediated interactions can change the effect of the same functional group. Furthermore, the final CA IX/CA XII classifier was an RBF-SVC operating on a combined Morgan/RDKit/Mordred feature space. Its KernelSHAP explanation, therefore, summarizes a nonlinear decision surface rather than a single additive SAR. The value of the structural comparison is consequently not that it proves a residue-level mechanism, but that it provides a coherent physical explanation for why the model repeatedly uses aromatic/hydrophobic versus polar local environments to distinguish the two tumor-associated isoenzymes.

CA II vs. CA IX showed intermediate but meaningful test performance (MCC = 0.459; balanced accuracy = 0.731; minority recall = 0.720). Unlike the two strongest models, the final classifier used only RDKit2D descriptors with logistic regression. Its SHAP profile was led by SMR_VSA4, followed by PEOE_VSA descriptors, ketone count, number of hydrogen-bond acceptors, additional molar-refractivity/VSA bins, and fragment counts, including aniline, halogen, and sulfonamide motifs (see Figure 4C). This pattern suggests that discrimination between CA II and CA IX is encoded more diffusely through the distribution of polarizability, partial-charge environments, hydrogen-bond acceptors, and fragment composition than through one dominant fingerprint environment.

Such a distributed signal is compatible with the comparatively subtle structural contrast between these isoforms. Important substitutions include Asn67/Gln67, Glu69/Thr69, Ile91/Leu91, Phe131/Val131, Val135/Leu135, and Leu204/Ala204 for CA II/CA IX, respectively [5]. Among these, position 131 has particularly strong experimental support. Crystal structures of ureido-substituted benzenesulfonamides showed that Phe131 in CA II can impose steric hindrance, whereas Val131 in CA IX and Ala131 in CA XII permit more favorable tail orientations [26]. Galati et al. independently linked ML-derived features for CA IX selectivity to X-ray structures and highlighted the same general principle: structurally important ligand substructures can be rationalized by their ability to adopt favorable orientations in the less obstructed CA IX environment relative to CA II [15]. The positive SHAP association of hydrogen-bond acceptor count and selected surface-area descriptors toward CA IX may, therefore, reflect the combined consequences of altered tail orientation, exposure to the hydrophilic half of the cavity, and water-mediated interactions rather than a single direct hydrogen bond.

The three CA-I-containing tasks were collectively less predictive, but the available evidence does not establish a single cause. Structural similarity is one plausible contributor, yet severe class imbalance, limited minority support, dataset size, chemotype composition, residual experimental variation, and model/split differences may also affect stability and generalization. For CA I vs. CA II, for example, the STRICT dataset contained only 23 first-isoenzyme-selective molecules (18 in outer TRAIN and 5 in outer TEST) against 775 second-isoenzyme-selective molecules, creating an extreme 33.7:1 imbalance (Supplementary Table S17). CA I and CA II also share the conserved catalytic zinc machinery, while several relevant differences lie toward the cavity entrance. CA I contains His67, Phe91, and Tyr204 instead of Asn67, Ile91, and Leu204 in CA II, and crystallographic work on CA I implicates the Leu198–Thr199–His200 loop and His67-containing water network in sulfonamide recognition [21]. The CA I/CA II SHAP map contained nitrogen-, sulfonamide-, ethynyl-aromatic-, and sulfur/oxygen-containing environments among the leading Morgan bits, but the TEST MCC confidence interval included zero and minority recall was only 0.20. Accordingly, these fragments should not be promoted to mechanistic design rules.

CA I vs. CA IX reached a modest but positive test MCC (0.308) with a bootstrap interval above zero, and its leading SHAP feature was the RDKit bicyclic-fragment count. A rigid or bicyclic scaffold could in principle interact differently with the more occluded CA I entrance and the more accessible CA-IX-selective region, but this model also had the lowest STRICT consensus AD coverage (66.3%; see Table 2), making chemical-space extrapolation a plausible contributor to reduced performance. The result is, therefore, better viewed as a weak but detectable pair-specific structure-selectivity signal rather than evidence for a general bicyclic CA IX pharmacophore.

CA I vs. CA XII is the most informative negative result. Despite a high-consensus AD coverage of 89.5% (see Table 2), the locked outer-test model was close to chance (MCC = 0.045; balanced accuracy = 0.530) and its MCC bootstrap interval crossed zero. The leading feature was the global Mordred descriptor BCUTd-1h rather than an easily localized fragment. This indicates that poor performance cannot be explained simply by most test compounds lying outside the modeled chemical domain. A more plausible interpretation is that, under the present 200/800 nM phenotype, the CA I/CA XII selectivity signal is heterogeneous relative to the available sample size and severe class imbalance and may require higher-order combinations of physicochemical and scaffold features that are not stable enough to generalize to the locked test set. Importantly, the significant Y-randomization result for this task does not contradict the poor test performance: Y-randomization shows that the development-set relationship is unlikely to be reproduced after random label permutation, whereas the outer test evaluates whether that learned relationship generalizes. The two analyses answer different questions, and the CA I/CA XII case illustrates why both are needed.

The STRICT/BROAD analysis further shows that selectivity performance is sensitive to the definition of the underlying evidence set. BROAD models improved over STRICT for the three CA-I-containing comparisons, but declined for CA II/IX, CA IX/XII, and especially CA II/XII. However, these differences cannot be interpreted as the isolated causal effect of requiring a shared source document. The end-to-end pipeline was re-optimized within each variant, so the active split, feature representation, and final algorithm sometimes changed between STRICT and BROAD. For example, the CA II/XII STRICT model used a random class-balanced split with Morgan2048/random forest, whereas the BROAD model used a scaffold-disjoint split with Morgan2048/logistic regression. The large MCC decrease in the BROAD pathway, therefore, demonstrates sensitivity of the complete data definition/model selection process, not a direct estimate of a provenance-filtering effect.

The AD and Y-randomization analyses provide complementary but limited evidence. High AD coverage means that a test molecule falls within the defined chemical similarity/leverage domain of TRAIN—it does not establish that its selectivity label is experimentally reliable or that the learned mapping is correct. The AD-IN/AD-OUT subgroup analysis supports this interpretation within the present dataset: CA I/CA XII remained near chance even within its large AD-IN subgroup, whereas some AD-OUT subsets were too small for stable metrics (Supplementary Table S18). Conversely, low AD coverage provides a reason for caution even when aggregate TEST performance is acceptable. All six STRICT models exceeded the 100 fixed-configuration randomized-label controls, but the observed comparator was selected through HPO, whereas the permuted runs were evaluated with the already selected hyperparameters and without threshold retuning. This asymmetry can favor the observed model, so the analysis should not be read as a fully exchangeable permutation test of the complete selection pipeline. The value 1/101 ≈ 0.00990 is also the resolution floor imposed by 100 permutations. In CA I/CA XII, the uniformly zero randomized MCC values together with macro-PR-AUC = 0.500 are consistent with degenerate constant-score/single-class predictions under the strongly regularized L1 logistic model, rather than with uniquely stronger biological signals. Y-randomization is, therefore, interpreted only as a fixed-configuration chance-correlation stress test, not as evidence of external generalizability.

A central purpose of the present analysis was to move beyond reporting model accuracy and identify ligand features associated with pairwise predictions. The comparison with crystallographic literature can provide chemical context, but it does not convert ligand-only SHAP attributions into protein–ligand interaction evidence. The 2D models contain no explicit protein coordinates, experimental binding poses, protonation microstates, ordered waters, or dynamics. Moreover, Morgan2048 is hash-folded, so one bit may represent multiple atom environments. Accordingly, a mapped Morgan fragment is only a representative structural realization of a model-associated bit, and agreement with an isoform-specific pocket substitution should be described as a structure-informed hypothesis rather than a residue-level mechanism. Matched cocrystal structures, experimentally constrained docking, mutagenesis, or prospective inhibition experiments would be required to establish direct protein–ligand interactions.

Several limitations should be considered when interpreting the findings. First, the binary phenotype deliberately excludes dual-active, dual-weak, and intermediate/mixed compounds, and the models, therefore, address selectivity direction only for the prespecified high-confidence subset rather than all CA ligands. Second, the 200/800 nM boundaries are operational modeling thresholds, not clinically validated cutoffs. They impose a minimum fourfold Ki separation while retaining enough compounds for modeling. Post hoc stricter-threshold analyses reduced cohort size substantially and did not remove the instability associated with sparse CA I minority classes (Supplementary Tables S14 and S15). Third, several datasets are highly imbalanced. In the STRICT data, the minority classes contained only 23 molecules for CA I/CA II and 37 for CA I/CA XII, and their TEST minority counts were 5 and 7, respectively (Supplementary Table S17). Class weighting and imbalance-aware metrics cannot compensate fully for such limited biological examples or guarantee stable bootstrap estimates. Fourth, the two highest point estimates were obtained from TEST cohorts of only 40 and 31 molecules for CA II/CA XII and CA IX/CA XII. Their bootstrap intervals quantify resampling uncertainty within these cohorts but do not eliminate sampling uncertainty arising from small independent TEST sets. Fifth, all bioactivity records ultimately derive from ChEMBL—the locked outer-TEST sets are held-out partitions, not independent external datasets from a separate source or prospective experiment. Temporal, independently literature-derived, or prospective biochemical validation is, therefore, still required. Sixth, same-document STRICT provenance reduces one component of between-publication heterogeneity but cannot harmonize assay pH, buffer, temperature, protein construct, substrate concentration, protocol, or laboratory effects that may influence Ki values and pairwise labels. Finally, the models use ligand-only 2D representations and do not directly model three-dimensional recognition, membrane access, cellular target engagement, pharmacokinetics, toxicity, or clinical benefit.

These distinctions are important for translation. The present evidence is computational and biochemical/preclinical: it concerns enzyme-level Ki-derived selectivity labels. A compound that is selective in an isolated-enzyme assay may fail to achieve isoenzyme-selective pharmacology in cells or patients because of permeability, extracellular versus intracellular target localization, protein expression, tissue exposure, metabolism, competing off-targets, or dose-limiting toxicity. Conversely, clinical utility cannot be inferred from a favorable pairwise classifier score. The models are, therefore, best viewed as hypothesis prioritization tools for medicinal chemistry and prospective biochemical testing, not as evidence of therapeutic efficacy.

These limitations also suggest clear future directions. Independent validation should be prioritized using temporally newer measurements, manually curated literature data not used for model development, or prospective biochemical testing with harmonized assay conditions. A controlled STRICT/BROAD comparison using fixed splits and fixed feature/model definitions could isolate the provenance component more directly. Structure-aware features derived from experimentally anchored protein–ligand complexes could complement the ligand-only models, and prospective testing of compounds selected from regions in which prediction, applicability domain, and SHAP evidence agree would provide the most decisive validation. The present six-pair framework can then serve as a benchmark for testing whether 3D interaction fingerprints, proteochemometric representations, or multitask learning improve selectivity prediction without sacrificing transparent phenotype definitions and locked-test safeguards.

## 4. Materials and Methods

### 4.1. Study Design and Target Selection

This study was designed as a pairwise molecular classification analysis of selectivity among four human carbonic anhydrase (CA) isoenzymes: carbonic anhydrase I (CA I; ChEMBL target ID CHEMBL261), carbonic anhydrase II (CA II; CHEMBL205), carbonic anhydrase IX (CA IX; CHEMBL3594), and carbonic anhydrase XII (CA XII; CHEMBL3242). The four targets generated six canonical pairwise classification tasks: CA I vs. CA II, CA I vs. CA IX, CA I vs. CA XII, CA II vs. CA IX, CA II vs. CA XII, and CA IX vs. CA XII (Supplementary Method S1).

The objective was not to classify compounds as universally “selective” or “nonselective.” Instead, a potency-constrained pairwise selectivity phenotype was defined to distinguish the direction of selectivity among compounds showing strong activity toward one isoenzyme and weak activity toward the paired isoenzyme. Activity thresholds, class definitions, split selection rules, model-ranking criteria, and the outer-test protection policy were fixed before final test-set inspection.

### 4.2. Retrieval and Assay-Level Curation of Experimental K_i_ Data

Experimental bioactivity records were retrieved independently for the four targets from the ChEMBL REST API on 18 August 2026 using ChEMBL release 37 (release date: 1 May 2026) [27]. The activity query was restricted to standard_type = Ki, and each ChEMBL target was verified as a Homo sapiens SINGLE PROTEIN target. Assay metadata were retrieved and linked to activity records through assay ChEMBL identifiers. The primary analysis used binding assays only (assay_type = B). The complete assay-level retention rules are reported in Supplementary Method S2.

Activity records were retained only when the target identifier matched the intended isoenzyme, the relation was exact (=), the standard value was positive and numeric, the unit could be converted to molar concentration (M, mM, μM/uM, nM, or pM), the data-validity comment was empty or indicated manual validation, the record was not flagged as a potential duplicate, standard_flag = 1 was required when available, the assay confidence score was at least 9, the BioAssay Ontology annotation corresponded to single-protein format, and the assay type was binding. Censored measurements were excluded. Ki values were converted to molar units and transformed as:

pK_i_ = −log_10_(K_i_ [M]).

The initial target-specific Ki queries yielded 11,944, 14,115, 7374, and 5618 activity rows for CA I, CA II, CA IX, and CA XII, respectively. Application of the binding-only assay and quality filters retained 5618, 6123, 5357, and 4419 activity rows, respectively. Document ChEMBL IDs, assay identifiers, confidence metadata, and other audit fields were retained throughout curation for provenance tracing. Target-level data flow and exact pairwise molecule flow are provided in Supplementary Tables S1 and S2.

### 4.3. Parent Mapping, Structure Standardization, and Molecule-Level Activity Aggregation

ChEMBL molecule metadata were retrieved for each curated activity record, and compounds were mapped to ChEMBL parent molecule identifiers. Parent canonical SMILES were preferentially used for structural processing; when a parent structure was unavailable, the molecule-level canonical SMILES served as a documented fallback. Molecular structures were processed with RDKit using parsing and sanitization, rdMolStandardize.Cleanup, FragmentParent extraction, removal of explicit hydrogens, and final sanitization. Stereochemical information and formal charge were preserved rather than automatically neutralized. The complete parent mapping and structure standardization sequence is given in Supplementary Method S3.

Invalid structures, structures without heavy atoms after standardization, and structures remaining multicomponent after parent-fragment processing were excluded. Canonical isomeric and non-isomeric SMILES and standardized InChIKeys were generated. Exact duplicate standardized structures were collapsed at the standardized isomeric canonical SMILES level, whereas stereoisomers were retained as distinct structures.

When multiple eligible Ki measurements mapped to the same standardized structure, the molecule-level observed activity was defined as the median pKi. The mean, standard deviation, minimum, maximum, interquartile range, number of measurements, number of assays, number of documents, and complete assay/document identifiers were retained as quality-control and provenance variables. Structures represented by at least two measurements with pKi standard deviation > 1.0 were flagged as having high experimental variability but were not automatically removed solely on this basis.

### 4.4. Physicochemical Filtering and Scaffold Annotation

For each standardized molecule, molecular weight, Wildman–Crippen logP, hydrogen-bond donor count, and hydrogen-bond acceptor count were calculated with RDKit. Classical Rule-of-Five limits were defined as molecular weight ≤ 500 Da, logP ≤ 5, hydrogen-bond donors ≤ 5, and hydrogen-bond acceptors ≤ 10 [28]. The molecule-level source used for selectivity analysis permitted a maximum of one Rule-of-Five violation, and the zero-violation set was retained for audit and sensitivity purposes rather than used as the primary filter. The exact Rule-of-Five audit criteria are summarized in Supplementary Method S4.

Bemis–Murcko scaffolds were calculated [29] as non-isomeric canonical scaffold SMILES. Acyclic molecules received unique scaffold-group identifiers instead of being pooled into a single generic acyclic group, thereby avoiding artificial grouping of unrelated acyclic structures during scaffold-aware partitioning. The acyclic-group fallback rule is documented in Supplementary Method S4.

### 4.5. Construction of BROAD and STRICT Pairwise Datasets

For each pair, the independently curated target-level molecule tables were joined using standardized InChIKey as the primary molecular identity key. Duplicate standardized InChIKeys in a purported molecule-level source were treated as a data-integrity error rather than silently aggregated, and standardized SMILES representations from the two target sources were cross-checked as an identity audit. Exact BROAD/STRICT eligibility logic and full sample-flow auditing are provided in Supplementary Methods S5 and S6 and Supplementary Tables S2 and S3.

The BROAD variant required only that the same standardized InChIKey was present in both isoenzyme datasets—the two target activity values were not required to originate from the same publication. The STRICT variant was a conservative provenance subset of BROAD: n_documents had to equal 1 for both target-specific molecule records, each record had to resolve to exactly one Document ChEMBL ID, and the two document identifiers had to be identical. STRICT was designated as the primary analysis, whereas BROAD was retained as a sensitivity/robustness analysis.

### 4.6. Locked Binary Selectivity Phenotype

For an ordered isoenzyme pair A vs. B, a compound was labeled A-selective when Ki(A) ≤ 200 nM and Ki(B) > 800 nM. Conversely, a compound was labeled B-selective when Ki(B) ≤ 200 nM and Ki(A) > 800 nM. These limits correspond approximately to pKi ≥ 6.699 for the active target and pKi < 6.097 for the weak paired target. Molecule-level median pKi values were converted back to Ki-equivalent concentrations solely for application of these prespecified thresholds. The complete category logic is reproduced in Supplementary Method S6.

The 200/800 nM thresholds were fixed before model training and were intended as an operational high-confidence definition rather than a universal pharmacological boundary. They require at least a fourfold Ki separation between the preferred and paired isoenzymes while retaining sufficient sample size for six pairwise tasks. CA medicinal chemistry studies typically report selectivity as a continuous selectivity index and use a wide range of potency/selectivity profiles rather than one accepted universal cutoff [18,19]. Therefore, threshold dependence was evaluated post hoc as a sensitivity analysis after the primary models had been frozen—the primary 200/800 nM models were not re-selected:

A-selective: K_i_(A) ≤ 200 nM AND K_i_(B) > 800 nM,

B-selective: K_i_(B) ≤ 200 nM AND K_i_(A) > 800 nM.

Binary label 0 represented selectivity toward the first isoenzyme named in the canonical pair, whereas label 1 represented selectivity toward the second. Compounds active against both isoenzymes (DUAL_ACTIVE), weak against both (DUAL_WEAK), or showing intermediate/mixed activity patterns were excluded from binary classifier training but retained in audit tables. Consequently, the models predict the direction of selectivity among molecules satisfying this high-confidence phenotype—they do not determine whether an arbitrary molecule is universally selective.

### 4.7. Molecular Representations and Leakage-Safe Preprocessing

Molecular features were generated once for the union of 1572 unique standardized molecules present across the final pairwise datasets. RDKit was used for core cheminformatics operations and calculation of 217 two-dimensional descriptors [30,31]. A separate Mordred-compatible feature store contained 1613 two-dimensional molecular descriptors. Mordred was developed as an open-source calculator covering a broad range of molecular descriptors for cheminformatics and QSAR/QSPR applications [32]. The third representation was a 2048-bit Morgan circular fingerprint calculated with radius 2, chirality included, and bond-type information. Morgan circular fingerprints are closely related to the extended-connectivity fingerprint (ECFP) family, in which atom-centered circular environments encode local molecular substructures for structure–activity modeling [33]. Seven logical feature spaces were evaluated: RDKit2D, Mordred2D, Morgan2048, RDKit2D+Mordred2D, Morgan2048+RDKit2D, Morgan2048+Mordred2D, and Morgan2048+RDKit2D+Mordred2D. Experimental activity values, assay/document identifiers, provenance fields, and quality-control variables were excluded from predictor features. Raw feature-store dimensions and the seven logical representations are detailed in Supplementary Method S7.

All preprocessing operations capable of learning from the data were fitted only within the corresponding training partition or cross-validation fold. Continuous descriptors were numerically coerced, infinities were converted to missing values, missing values were median-imputed using training data, training-constant variables were removed, and one member of highly correlated descriptor pairs with |r| ≥ 0.95 was removed. Morgan features remained binary—missing values were set to zero, training-constant bits were removed, and the fingerprint block was neither scaled nor Pearson correlation filtered. For logistic regression and RBF-SVC, standardization was applied only to the continuous descriptor block using training-derived parameters.

### 4.8. Outer Partitioning, Active-Split Selection, and Model Screening

Three candidate 80:20 outer partitioning strategies were generated independently for each pairwise dataset: (i) stratified random splitting, (ii) a class-wise farthest-first/maxi–min split based on Jaccard distance between binary Morgan fingerprints, conceptually related to Kennard–Stone representative sampling [34], and (iii) a scaffold-disjoint balanced split based on Bemis–Murcko scaffold groups [29]. For the scaffold strategy, randomized group searches were used to identify a partition approximating the target test fraction while preserving both classes and maintaining scaffold disjointness. Exact candidate-split algorithms, selector scores for all three candidate splits, and the selected active split for every STRICT/BROAD dataset are reported in Supplementary Methods S8 and S9 and Supplementary Table S4.

No candidate outer-test model performance was used to choose the split. Instead, each candidate split was evaluated only within its training subset using a fixed selector consisting of Morgan2048 fingerprints and a class-weighted ExtraTrees classifier with 250 trees. Threefold cross-validation was used, and the active split was selected by the highest mean MCC, with macro-PR-AUC and balanced accuracy as sequential tiebreakers. The selected outer-test partition was then locked. Exactly three candidate outer-split strategies were considered per dataset. Because the strategy itself was chosen from TRAIN-only CV diagnostics, the resulting TEST partition remained untouched by model scoring, but its composition was data-dependent; accordingly, the final TEST estimate is a locked held-out evaluation rather than an independent external validation estimate.

Lightweight screening was performed only within the selected outer-training data using the seven feature spaces and four classifier families: ExtraTrees, random forest, class-weighted logistic regression, and class-weighted radial basis function support vector classification (RBF-SVC). Here, “lightweight screening” denotes a deliberately limited, computationally economical pre-HPO comparison used to identify promising and methodologically diverse feature/model combinations—it was not treated as final model selection. Support vector machines construct a maximum-margin decision boundary in a transformed feature space [35]. For the RBF-SVC models, the Gaussian radial basis kernel was K(x_i_, x_j_) = exp(−γ||x_i_ − x_j_||^2^), allowing nonlinear separation. Class weighting was used to reduce majority-class dominance: with the balanced option, the class-specific weight is inversely proportional to class frequency, w_c = n/(K·n_c), where n is the number of training samples, K is the number of classes, and n_c is the number of samples in class c. The implementations were provided by scikit-learn [36]. Synthetic oversampling, including SMOTE, was not used. Candidate combinations were ranked principally by MCC, then macro-PR-AUC and balanced accuracy, and a diversity-aware subset was passed to full hyperparameter optimization.

### 4.9. Hyperparameter Optimization, Global Model Lock, and Decision Threshold Selection

Full hyperparameter optimization (full-HPO) was conducted exclusively on the locked outer-training data. In this study, “full-HPO” denotes the second-stage, higher-resolution optimization performed only after lightweight screening, using the prespecified search spaces of the retained candidate model families. It is, therefore, an operational term for the complete train-only optimization stage rather than the name of a separate machine learning algorithm. Here, 4 diversity-aware feature/model candidate combinations were retained per dataset, and 12 configurations were evaluated per candidate, yielding up to 48 full-HPO trials per STRICT/BROAD dataset. The target cross-validation design used five folds. StratifiedKFold partitions samples into non-overlapping folds while approximately preserving class proportions in each fold. For scaffold-disjoint active splits, StratifiedGroupKFold was used instead: it additionally keeps each scaffold group entirely within a single fold, preventing the same scaffold group from appearing simultaneously in a training and validation subset while attempting to preserve class balance. Both splitting procedures were implemented in scikit-learn [36]. Fold-specific preprocessing was repeated independently inside every cross-validation fold. Configurations were ranked by mean CV-MCC, followed by macro-PR-AUC and balanced accuracy. Full parameter spaces, candidate counts, and per-dataset winning configurations are reported in Supplementary Methods S10 and S11 and Supplementary Table S5.

For ExtraTrees, the sampled search considered 500–1200 estimators, max_features values of sqrt or 0.20–0.70, maximum depths from 10 to 32 or unrestricted depth, minimum split sizes of 2–12, minimum leaf sizes of 1–5, bootstrap on/off, and gini/entropy criteria. Random forest used analogous tree-depth and node-size ranges with max_features of sqrt or 0.20–0.50 and bootstrap enabled. Logistic regression evaluated C values from 0.005 to 100 with L1/L2 penalties using liblinear, and RBF-SVC evaluated C values from 0.1 to 100 and gamma values from 0.0003 to 0.1 plus the scale setting. Class weighting was balanced throughout. A compatible minimal-development configuration could be included as a deterministic seeded candidate in the full-HPO plan, so a final winner was not required to coincide only with the sampled grid levels.

A global model-locking step prevented sequential test-set feedback: HPO and winner selection were completed for all 12 datasets (6 pairs × STRICT/BROAD) before any final outer-test metric was inspected. Only after all winners were frozen was final test evaluation permitted.

For reproducibility, the six primary OOF-selected thresholds and their OOF MCC values are now reported in Supplementary Table S16. The model-default cutoff was 0.5 for probability-like scores and 0 for the RBF-SVC decision function. No OOF threshold was selected using outer-TEST predictions.

For each locked winner, fivefold out-of-fold (OOF) scores were generated on the complete outer-training data. The operating threshold was selected from these OOF scores by maximizing MCC, with balanced accuracy and macro-F1 used as secondary criteria and proximity to the model default used as a final tiebreaker. The outer-test set was never used for threshold selection. The OOF-derived threshold was frozen before the one-time final test evaluation.

### 4.10. Performance Metrics and Uncertainty

Because several pairwise datasets were markedly imbalanced, the Matthews correlation coefficient (MCC) was designated the primary performance metric. MCC uses all four cells of the binary confusion matrix and is commonly recommended as a comparatively informative single-score summary when both classes are important [37]. Precision–recall analysis was retained because PR-based evaluation is particularly informative under class imbalance [38]. Additional measures included balanced accuracy, macro-F1, class-specific precision and recall, minority-class recall, ROC-AUC, confusion matrices, and macro-PR-AUC. Let TP, TN, FP, and FN denote true positives, true negatives, false positives, and false negatives, respectively. The main reported metrics were defined as follows:

MCC = (TP·TN − FP·FN)/√[(TP + FP)(TP + FN)(TN + FP)(TN + FN)],

Recall (sensitivity) = TP/(TP + FN),

Specificity = TN/(TN + FP),

Balanced accuracy = ½(Recall + Specificity),

Precision = TP/(TP + FP),

F1 = 2·(Precision·Recall)/(Precision + Recall); Macro-F1 = ½(F1_0_ + F1_1_),

AP = Σ_n_ (R_n_ − R_n−1_)P_n_; Macro-PR-AUC = ½(AP_0_ + AP_1_),

ROC-AUC = ∫_0_^1^ TPR(FPR) d(FPR).

Minority-class recall was the recall calculated for the less frequent class in the corresponding dataset. For macro-PR-AUC, average precision (AP) was calculated separately after treating class 0 and class 1 as the positive class in turn, and the two AP values were averaged so that the result did not privilege the arbitrary label-1 orientation. The AP expression above follows the standard stepwise precision–recall formulation used in scikit-learn [36,38].

Following the global model lock, each final outer-test set was evaluated once. Uncertainty of MCC, macro-PR-AUC, balanced accuracy, macro-F1, and minority-class recall was estimated using 1000 stratified bootstrap resamples. For a metric θ, the percentile interval was defined as CI_95_% = [Q_0_._025_(θ*), Q_0_._975_(θ*)], where θ* denotes the metric calculated in a bootstrap resample. Thus, the 2.5th and 97.5th percentiles of the bootstrap distribution were used as the 95% confidence interval limits. Full locked outer-test metrics for all 12 final STRICT/BROAD models are provided in Supplementary Table S6, and bootstrap details are given in Supplementary Method S12.

### 4.11. Applicability Domain, Y-Randomization, and Model Interpretation

The applicability domain (AD) was evaluated for all 12 final models using complementary structural similarity and leverage criteria. For the Tanimoto structural similarity AD [39], the 5th percentile of the training leave-one-out nearest-neighbor Morgan similarity distribution was used as the threshold. A test compound was classified as Tanimoto-AD-IN when its maximum similarity to the training set met or exceeded this threshold. For the leverage AD, the final processed feature space was standardized using training data only and projected by principal component analysis to approximately 95% explained variance, with a maximum of 30 components. The leverage warning limit was h* = 3(p + 1)/ntrain [40]. Consensus AD-IN required both Tanimoto-AD-IN and PCA-leverage-AD-IN. Standardized residual criteria were not used because the endpoint was categorical. Complete AD results are reported in Supplementary Method S13, Supplementary Table S8, and Supplementary Figure S3. Consensus AD was defined as a chemical-space diagnostic and was not intended to quantify the reliability of experimental selectivity labels. Final frozen TEST predictions were additionally summarized within AD-IN and AD-OUT subgroups without retraining (Supplementary Table S18).

Chance correlation was assessed for the six STRICT primary models using 100 Y-randomization permutations per model. The final feature representation, classifier family, optimized hyperparameters, and cross-validation family were kept fixed—HPO was not repeated and classification thresholds were not re-optimized. The observed comparator was the CV-MCC of the HPO-selected final configuration, whereas each permuted-label run used that already selected configuration. Consequently, this protocol is a fixed-configuration response-randomization stress test and is not a fully exchangeable permutation test of the complete HPO/model selection procedure—the asymmetry may favor the observed model. The empirical exceedance value compared the observed locked CV-MCC with the randomized-label MCC distribution using the response-randomization procedure described in Supplementary Method S14 [41]. With Nperm = 100, the minimum attainable value under this correction is 1/101 = 0.00990—this is a permutation-resolution limit rather than a precise tail-probability estimate:

p_emp_ = [1 + #(MCCrandom ≥ MCCobserved)]/(Nperm + 1).

SHapley Additive exPlanations (SHAP) were applied to the six STRICT primary models. SHAP is a game-theoretic feature-attribution framework that assigns additive contribution values to individual features for a model prediction [42]. TreeExplainer was used for tree ensembles, LinearExplainer for logistic regression, and a computationally bounded KernelExplainer for RBF-SVC. Global importance was summarized by mean absolute SHAP value, and mean signed SHAP was retained to indicate the direction of contribution. For Morgan-containing models, highly ranked fingerprint bits were mapped to representative atom-centered molecular environments and exported as fragment SMILES and highlighted molecular images. Because hashed Morgan fingerprints can map more than one atom environment to the same bit, the exported fragments were treated as representative structural realizations rather than unique definitions of a bit. SHAP-derived features were interpreted as model-associated structural correlates rather than isolated mechanistic causes. Explainer-specific settings and primary model summaries are provided in Supplementary Method S15 and Supplementary Tables S10 and S11, and complementary CA-I-containing SHAP profiles are shown in Supplementary Figures S4–S6.

### 4.12. Post Hoc Sensitivity and Subgroup Analyses

All post hoc sensitivity and subgroup analyses were conducted after the primary models, splits, hyperparameters, and outer-TEST predictions had been frozen. They were not used to re-select any primary model. The analyses comprised (i) exact STRICT cohort counts under alternative thresholds of 100/800, 200/1000, 100/1000, and 300/600 nM, (ii) descriptive performance of the existing locked TEST predictions within nested stricter-threshold subsets, (iii) OOF performance at model-default and pre-TEST tuned thresholds, (iv) explicit TRAIN/TEST minority-class support, (v) performance stratified by consensus AD-IN/AD-OUT status, and (vi) a residual activity-heterogeneity audit using retained measurement counts, assay counts, molecule-level pKi standard deviations, and variability flags. Full results are provided in Supplementary Tables S14–S19 and Supplementary Figures S7 and S8.

### 4.13. Reproducibility and Computational Safeguards

The workflow was implemented primarily in Google Colab. Database retrieval and structure standardization manifests record Python 3.12.13, pandas 2.2.3, NumPy 2.0.2, RDKit 2026.03.5, and Matplotlib 3.10.0. RDKit is an open-source cheminformatics toolkit widely used for molecular standardization, descriptors, fingerprints, and scaffold operations [30,31]. Frozen estimators were serialized under scikit-learn 1.6.1, which provided the machine learning estimators, cross-validation splitters, preprocessing routines, and evaluation utilities [36]. Mordred-compatible 2D descriptors were generated with mordredcommunity 2.0.7, following the descriptor definitions introduced for Mordred [32], and SHAP 0.52.0 was used for model interpretation [42]. The principal project random seed was 42. Publication-run software/database provenance is summarized in Supplementary Table S13.

Each major analysis stage generated machine-readable manifests containing dataset definitions, thresholds, input/output paths, and hashes. Critical datasets and final estimators were protected by SHA-256 checks. Following final model generation, all 12 saved STRICT/BROAD estimators were independently reloaded together with their saved preprocessed test matrices. Score vectors, OOF-derived threshold predictions, class labels, and final metrics were regenerated and compared with stored outputs before downstream interpretation. The independent 12-model reproducibility audit is reported in Supplementary Method S16 and Supplementary Table S12.

### 4.14. Generative Artificial Intelligence Disclosure

Generative artificial intelligence tools, including ChatGPT 5.6 (OpenAI), were used to assist with code drafting, workflow documentation, literature-search organization, and language editing during manuscript preparation. All data-curation rules, model definitions, executable code, numerical outputs, citations, and scientific interpretations were reviewed and verified by the authors. All numerical results reported in this article originate from the executed and archived computational workflows rather than from generative-AI estimates.

## 5. Conclusions

The principal conclusion of this study is that the six CA pairwise selectivity tasks display different levels of predictive separability within the present ChEMBL-derived datasets and locked validation framework. The highest observed held-out scores occurred for CA II/CA XII and CA IX/CA XII, CA II/CA IX was intermediate, and CA-I-containing comparisons were less stable, particularly where minority support was sparse. Because the two highest point estimates were obtained from TEST cohorts of only 40 and 31 compounds, respectively, they should be regarded as promising pair-specific signals that warrant independent external or prospective validation rather than as definitive evidence of broad generalizability. These differences should not be interpreted as an intrinsic biological ranking of isoenzyme “learnability.” SHAP patterns were chemically compatible with known active-site differences, but they remain ligand-based, model-associated hypotheses rather than direct evidence of residue-level interactions. By combining a fixed potency-constrained phenotype with provenance-aware datasets, locked outer testing, Y-randomization, applicability domain analysis, bootstrap uncertainty, reproducibility checks, and post hoc sensitivity analyses, the study provides a transparent framework for identifying promising CA selectivity signals while also defining where current data are insufficient for strong generalization claims. Independent external or prospective validation remains necessary.

## Figures and Tables

**Table 1:** Pairwise dataset composition and class balance. STRICT was the primary analysis, and BROAD was retained for sensitivity analysis.

Pair	BROAD N	STRICT N	STRICT Class 0	STRICT Class 1	Minority N	Maj./Min. Ratio	STRICT Retention (%)
CA I vs. CA II	837	798	23	775	23	33.70	95.3
CA I vs. CA IX	932	905	89	816	89	9.17	97.1
CA I vs. CA XII	802	761	37	724	37	19.57	94.9
CA II vs. CA IX	319	300	176	124	124	1.42	94.0
CA II vs. CA XII	232	200	55	145	55	2.64	86.2
CA IX vs. CA XII	161	152	52	100	52	1.92	94.4

Class 0 denotes selectivity toward the first isoenzyme named in the pair, and class 1 denotes selectivity toward the second. Minority N and the majority/minority ratio refer to the STRICT dataset. STRICT retention = 100 × STRICT N/BROAD N.

**Table 2:** Final configuration and performance of the six primary STRICT pairwise selectivity models. MCC confidence intervals are 95% percentile intervals from 1000 stratified bootstrap resamples of the locked outer-test sets.

Pair	Outer Split	Feature Set	Classifier	Test N	CV MCC	TEST MCC (95% CI)	Macro-PR-AUC	Balanced Accuracy	Macro-F1	Minority Recall	Consensus AD-IN
CA I vs. CA II	Random	Morgan+RDKit	ExtraTrees	160	0.492	0.303 (−0.025–0.661)	0.624	0.597	0.635	0.200	86.2%
CA I vs. CA IX	Scaffold	Morgan+RDKit	LogReg	181	0.444	0.308 (0.092–0.514)	0.638	0.658	0.654	0.389	66.3%
CA I vs. CA XII	Scaffold	Mordred2D	LogReg	152	0.422	0.045 (−0.075–0.302)	0.515	0.530	0.518	0.143	89.5%
CA II vs. CA IX	Scaffold	RDKit2D	LogReg	60	0.538	0.459 (0.245–0.690)	0.768	0.731	0.729	0.720	75.0%
CA II vs. CA XII	Random	Morgan2048	RF	40	0.695	0.748 (0.541–0.937)	0.991	0.818	0.857	0.636	90.0%
CA IX vs. CA XII	Random	Morgan+RDKit+Mordred	RBF-SVC	31	0.667	0.718 (0.436–0.933)	0.933	0.859	0.859	0.818	93.5%

CV MCC is the locked full-HPO mean cross-validated MCC on outer TRAIN. TEST metrics use the OOF-selected decision threshold. Consensus AD-IN requires both the Tanimoto similarity and PCA-leverage criteria.

**Table 3:** Selected amino acid differences in and around the active-site selectivity region of CA I, CA II, CA IX, and CA XII.

Position *	CA I	CA II	CA IX	CA XII	Selectivity-Relevant Physicochemical Contrast
60	Ile	Leu	Arg	Thr	Hydrophobic in CA I/II; positively charged in CA IX; polar in CA XII
62	Val	Asn	Asn	Asn	CA I uniquely more hydrophobic at this position
65	Ser	Ala	Ser	Ser	Small polar residue in CA I/CA IX/CA XII versus Ala in CA II
67	His	Asn	Gln	Lys	Large change in H-bonding/charge toward the cavity exit
69	Asn	Glu	Thr	Asn	Acidic in CA II; neutral polar residues in CA I/CA IX/CA XII
91	Phe	Ile	Leu	Thr	Bulky aromatic in CA I; hydrophobic in CA II/CA IX; polar in CA XII
131	Leu	Phe	Val	Ala	Progressively decreasing side-chain bulk; Phe131 in CA II is a major steric determinant
135	Ala	Val	Leu	Ser	Hydrophobicity increases toward CA IX but becomes polar in CA XII
204	Tyr	Leu	Ala	Asn	Bulky aromatic in CA I; hydrophobic/small in CA II/CA IX; polar in CA XII

* CA II numbering is used for cross-isoform comparison, following the aligned residue map reported by Singh et al. [5]. Because PDB constructs and publications can use different sequence/construct numbering conventions, residue identities should be checked against the exact experimental structure before structure-specific modeling.

## Data Availability

The curated pairwise datasets, English-language analysis notebooks, and machine-readable summary outputs supporting this study are publicly available at: https://github.com/ErolEroglu/CA-Izoenzymes-Selectivity-ML. Source bioactivity data were retrieved from ChEMBL release 37.

## Supplementary Materials

The following supporting information can be downloaded at: https://www.mdpi.com/article/10.3390/ijms27198784/s1.

## Abbreviations

The following abbreviations are used in this manuscript:

**Abbreviation**	**Definition**
AD	Applicability domain
AP	Average precision
CA	Carbonic anhydrase
CAI	Carbonic anhydrase inhibitor
CV	Cross-validation
ECFP	Extended-connectivity fingerprint
HPO	Hyperparameter optimization
MCC	Matthews correlation coefficient
ML	Machine learning
OOF	Out-of-fold
PCA	Principal component analysis
PR-AUC	Area under the precision–recall curve
QSAR	Quantitative structure–activity relationship
RBF-SVC	Radial basis function support vector classifier
RF	Random forest
SHAP	SHapley Additive exPlanations
