# Supplementary material for: Machine Learning-Based Classification of Pairwise Selectivity Among Human Carbonic Anhydrase I, II, IX, and XII Isoenzymes
Source: Int J Mol Sci. 2026 Sep 30;27(19):8784. doi: 10.3390/ijms27198784 (PMC13664808; doi:10.3390/ijms27198784)
Supplement: Supplementary file 1 [file ijms-27-08784-s001.zip › ijms-4568494-supplementary.pdf]

Supplementary Material

Machine Learning-Based Classification of Pairwise Selectivity among Human Carbonic Anhydrase I, II, IX, and XII Isoenzymes

Supplementary Methods, Tables, Figures, and Reproducibility Documentation

Frozen publication-stage data: ChEMBL release 37; data retrieval 18 August 2026
Supplementary compilation date: 27 August 2026

Scope. This Supplementary Material supports the classification study only. It contains the operational curation rules, pairwise data flow, frozen split/model-selection safeguards, final STRICT/BROAD model summaries, applicability-domain results, 100-permutation Y-randomization, SHAP interpretation, and reproducibility audits. Binding-affinity regression analyses are outside the scope of this manuscript.

Primary versus sensitivity analysis. STRICT is the primary analysis. BROAD is an end-to-end sensitivity analysis. Because the STRICT and BROAD pipelines were optimized independently, differences in their final performance are descriptive sensitivity results and must not be interpreted as isolated causal effects of document provenance.

Label orientation. In every ordered pair A vs B, binary label 0 denotes selectivity toward the first isoenzyme and label 1 denotes selectivity toward the second. The frozen phenotype requires Ki ≤200 nM at the selected target and Ki >800 nM at the paired off-target.

Machine-readable companion files. The GitHub-ready repository archive accompanying this manuscript contains the frozen target snapshots, all 12 final pairwise datasets, stage-level central summaries, English publication notebooks, and SHA-256 file manifest. The CSV table files underlying Supplementary Tables S1–S13 are retained separately in the repository.

Supplementary Methods

References cited in the Supplementary Methods use the same numbering as the main manuscript; relevant bibliographic entries are reproduced at the end of this document.

Supplementary Method S1. Target registry and pairwise task definition

The analysis considered four Homo sapiens carbonic anhydrase single-protein targets: CA I (CHEMBL261), CA II (CHEMBL205), CA IX (CHEMBL3594), and CA XII (CHEMBL3242). These targets generated six canonical ordered pairwise tasks: CA I vs CA II, CA I vs CA IX, CA I vs CA XII, CA II vs CA IX, CA II vs CA XII, and CA IX vs CA XII. Target-level source counts are summarized in Supplementary Table S1.

The study endpoint is a potency-constrained pairwise selectivity phenotype, not a universal selective/non-selective label. Thresholds and label orientation were frozen before model screening. For A vs B, label 0 = A-selective and label 1 = B-selective.

Supplementary Method S2. Exact ChEMBL activity-curation logic

Experimental bioactivity records were retrieved independently for the four targets through the ChEMBL REST API on 18 August 2026 using ChEMBL release 37 [28]. The primary selectivity source used exact Ki measurements from binding assays. The following filters were applied before parent mapping and molecule-level aggregation:

- Target identifier had to match the intended isoenzyme, and each target was verified as Homo sapiens SINGLE PROTEIN.
- standard_type = Ki and relation = "=" were required; censored measurements were excluded.
- standard_value had to be positive and numeric.
- Units had to be convertible to molar concentration: M, mM, µM/uM, nM, or pM.
- data_validity_comment had to be empty or indicate manual validation.
- Records flagged as potential duplicates were excluded.
- standard_flag = 1 was required when the field was available.
- Assay confidence score had to be at least 9.
- The BioAssay Ontology annotation had to correspond to a single-protein format.
- assay_type = B (binding) was required for the primary selectivity analysis.
- Document ChEMBL IDs, assay ChEMBL IDs, confidence metadata, and other audit fields were retained for provenance tracing.

$$pK_i = -\log_{10}(K_i\ [M])$$

Initial query counts, post-assay-quality counts, and final target-level molecule counts are reported in Supplementary Table S1.

Supplementary Method S3. Parent mapping, structure standardization, and molecule-level aggregation

- ChEMBL molecule metadata were retrieved for each activity molecule ID, including canonical SMILES and molecular hierarchy. The hierarchy parent_chembl_id was used when available; otherwise the queried molecule ID served as its own parent identifier.
- Parent molecule metadata were retrieved separately. parent_canonical_smiles was the preferred structural input; molecule_canonical_smiles served as fallback when the parent structure was unavailable.
- RDKit processing consisted of MolFromSmiles(sanitize=True), rdMolStandardize.Cleanup, rdMolStandardize.FragmentParent, RemoveHs, and Chem.SanitizeMol [1,2].
- Structures without heavy atoms, invalid structures, and structures remaining multicomponent after standardization were excluded.
- Canonical isomeric and non-isomeric SMILES, standardized InChIKey, fragment count, heavy-atom count, molecular weight, formal charge, and stereocenter count were recorded. Stereochemistry and formal charge were preserved.
- Molecule-level aggregation was performed at the standardized isomeric canonical-SMILES level. The median pKi was used as observed activity; mean, SD, minimum, maximum, 25th/75th percentiles, range, and IQR were retained.

- Provenance fields included the number and identities of assays, molecules, parent IDs, documents, and activity records. pKi SD >0.5 was flagged as moderate variability and >1.0 as high variability; neither flag caused automatic exclusion.
- Non-isomeric SMILES groups containing multiple isomeric structures were retained for audit rather than collapsed, preserving stereoisomers as distinct standardized structures.

Supplementary Method S4. Physicochemical filtering and scaffold audit

The molecule-level selectivity source allowed at most one violation of the classical Rule-of-Five limits: molecular weight ≤500 Da, Wildman–Crippen logP ≤5, H-bond donors ≤5, and H-bond acceptors ≤10 [29]. Equality with a boundary was compliant; only values above the boundary counted as violations. The zero-violation set was retained for audit/sensitivity purposes rather than used as the primary selectivity source.

Audit-only quantities included TPSA, rotatable bonds, heavy atoms, ring count, aromatic ring count, fraction sp3, and formal charge. Bemis–Murcko scaffolds were generated as non-isomeric canonical scaffold SMILES [31]. Acyclic compounds were assigned unique ACYCLIC_<identifier>-style scaffold groups rather than pooled into a single generic group, preventing unrelated acyclic molecules from being forced into one scaffold group during scaffold-aware partitioning.

Supplementary Method S5. Pairwise matching, identity checks, and sample-flow audit

For each pair, target-level molecule tables were joined by standardized InChIKey. Duplicate standardized InChIKeys in a purported molecule-level source were treated as a data-integrity error rather than silently aggregated. Standardized SMILES from the two target-specific sources were also compared as a representation-consistency audit.

"Common molecules" in Supplementary Table S2 are the pairwise InChIKey intersections for BROAD or the corresponding provenance-filtered STRICT subset. Binary N equals first-isoenzyme-selective plus second-isoenzyme-selective molecules. DUAL_ACTIVE, DUAL_WEAK, and INTERMEDIATE_OR_MIXED molecules remain in the audit but are excluded from classifier training. Final class balance and integrity checks are reported in Supplementary Table S3.

Supplementary Method S6. BROAD/STRICT definitions and locked binary phenotype

BROAD eligibility required a shared standardized InChIKey in the two target-specific molecule-level sources. STRICT eligibility required BROAD eligibility plus n_documents = 1 for both targets, exactly one parsed Document ChEMBL ID on each target-specific record, and equality of those two document IDs. Document identifiers were normalized to unique uppercase CHEMBL tokens before comparison.

For an ordered pair A vs B, the mutually exclusive audit categories were assigned from Ki-equivalent values as follows:

- A_SELECTIVE / label 0: Ki(A) ≤200 nM and Ki(B) >800 nM.
- B_SELECTIVE / label 1: Ki(B) ≤200 nM and Ki(A) >800 nM.
- DUAL_ACTIVE: both Ki values ≤200 nM.
- DUAL_WEAK: both Ki values >800 nM.
- INTERMEDIATE_OR_MIXED: all remaining patterns.

Only A_SELECTIVE and B_SELECTIVE entered binary training. The thresholds correspond approximately to pKi ≥6.699 for the active target and pKi <6.097 for the weak/off-target. A compound active at both targets is DUAL_ACTIVE even if one target is substantially more potent; the phenotype is therefore intentionally more stringent than a generic fold-selectivity definition.

Supplementary Method S7. Central feature store and logical molecular representations

Features were generated once for the union of 1,572 unique standardized molecules and were referenced by standardized InChIKey rather than recomputed for each pair. Activity, provenance, assay, document, and QC variables were excluded from predictors.

- RDKit2D: 217 two-dimensional descriptors [1,2].
- Mordred2D: 1,613 two-dimensional descriptors generated with the Mordred-compatible mordredcommunity package, following Mordred descriptor definitions [3].

- Morgan2048: 2,048-bit Morgan circular fingerprint, radius 2, chirality included, bond-type information included [4].
- Seven logical feature spaces: RDKit2D; Mordred2D; Morgan2048; RDKit2D+Mordred2D; Morgan2048+RDKit2D; Morgan2048+Mordred2D; Morgan2048+RDKit2D+Mordred2D.

All data-dependent preprocessing was deferred to training partitions/folds. Continuous descriptors were numerically coerced, median-imputed from TRAIN, stripped of TRAIN-constant variables, and correlation-filtered so one variable from descriptor pairs with $|r| \geq 0.95$ was removed. Morgan bits remained binary; only TRAIN-constant bits were removed. For logistic regression and RBF-SVC, standardization was applied only to continuous descriptor blocks.

Supplementary Method S8. Candidate outer splits and TRAIN-only active-split selection

Three 80:20 candidate outer partitions were created independently for every STRICT/BROAD dataset:

- Stratified random: an 80:20 class-balanced random split.
- Class-wise maximin/KS-like: farthest-first representative TRAIN selection in Morgan/Jaccard geometry, conceptually related to Kennard–Stone sampling [30], performed separately within classes to preserve representation of both labels.
- Scaffold-disjoint balanced: Bemis–Murcko groups were kept disjoint between TRAIN and TEST; randomized group searches sought an approximately 20% TEST fraction while retaining both classes and reasonable balance.

A fixed selector (Morgan2048 + ExtraTreesClassifier, 250 estimators, max_features="sqrt", class_weight="balanced") was evaluated by three-fold StratifiedKFold within each candidate outer TRAIN partition. Candidate splits were ranked by mean CV-MCC, then macro PR-AUC, then balanced accuracy. Candidate outer TEST performance was never evaluated during split selection. Selector scores for all three candidate outer splits and the selected active split are reported for every STRICT/BROAD dataset in Supplementary Table S4.

Supplementary Method S9. Lightweight classifier screening

Within the selected outer TRAIN only, the seven logical feature spaces were screened with ExtraTreesClassifier, RandomForestClassifier, class-weighted LogisticRegression, and class-weighted RBF-SVC. Synthetic oversampling, including SMOTE, was not used. If the active outer split was scaffold-disjoint, the inner screening split was also scaffold-group-disjoint; otherwise a stratified random inner validation split was used. Candidate combinations were ranked by MCC, macro PR-AUC, and balanced accuracy. A diversity-aware subset across feature spaces and model families was forwarded to full HPO.

Supplementary Method S10. Full hyperparameter-optimization search spaces

For each STRICT/BROAD dataset, four diversity-aware feature/model candidates were passed to full HPO. Twelve deterministic ParameterSampler configurations were evaluated per candidate, yielding up to 48 trials per dataset. Five-fold CV was targeted; scaffold-disjoint active splits used StratifiedGroupKFold, while other active splits used StratifiedKFold [5]. Preprocessing was refit independently within each CV fold.

- ExtraTrees: 500–1,200 estimators; max_features = sqrt or 0.20–0.70; max_depth = 10–32 or None; min_samples_split = 2–12; min_samples_leaf = 1–5; bootstrap on/off; gini/entropy criteria.
- Random Forest: analogous depth/node-size ranges; max_features = sqrt or 0.20–0.50; bootstrap enabled.
- Logistic regression: C = 0.005–100; L1/L2 penalties with liblinear; class_weight = balanced.
- RBF-SVC: C = 0.1–100; gamma = 0.0003–0.1 plus "scale"; class_weight = balanced [6].
- A compatible minimal-development configuration could be included as a deterministic seeded candidate; the final winner was selected only from locked outer TRAIN results.

Final HPO ranking was mean CV-MCC, then macro PR-AUC, then balanced accuracy. Per-dataset winners are reported in Supplementary Table S5.

Supplementary Method S11. Global winner lock, OOF threshold selection, and final TEST protocol

A GLOBAL_HPO_LOCK was created only after all 12 HPO searches were complete. This prevented sequential feedback from early outer TEST results into later model-selection decisions. Only after all 12 winners were frozen could any final outer TEST evaluation proceed.

Each winner was re-evaluated by five-fold OOF prediction on its complete outer TRAIN data. Candidate operating thresholds consisted of the model default plus thresholds drawn from 199 score quantiles spanning the 1st to 99th percentiles. Thresholds were ranked by MCC, then balanced accuracy, macro F1, and finally proximity to the default threshold. The selected threshold was frozen before TEST evaluation. The one-time locked TEST results are summarized in Supplementary Table S6.

Supplementary Method S12. Classification metrics and bootstrap uncertainty

MCC was the primary metric because it uses all four confusion-matrix cells and is especially useful for imbalanced binary classification [7]. Macro PR-AUC was used because precision–recall evaluation is informative under class imbalance [8] and was computed symmetrically across the two label orientations.

$$\mathrm{MCC} = (\mathrm{TP}\cdot\mathrm{TN} - \mathrm{FP}\cdot\mathrm{FN}) / \sqrt{[(\mathrm{TP}+\mathrm{FP})(\mathrm{TP}+\mathrm{FN})(\mathrm{TN}+\mathrm{FP})(\mathrm{TN}+\mathrm{FN})]}$$

$$\text{Balanced accuracy} = \tfrac{1}{2}[\text{Sensitivity} + \text{Specificity}]$$

$$\mathrm{F1} = 2\cdot\text{Precision}\cdot\text{Recall} / (\text{Precision} + \text{Recall}); \quad \text{Macro-F1} = \tfrac{1}{2}(\mathrm{F1}_0 + \mathrm{F1}_1)$$

$$\text{Macro PR-AUC} = \tfrac{1}{2}(\text{APclass } 0 + \text{APclass } 1)$$

Additional outputs included ROC-AUC, class-specific precision/recall, minority-class precision/recall, accuracy, and confusion matrices. Final TEST uncertainty was estimated with 1,000 stratified bootstrap resamples: samples were drawn with replacement separately within class 0 and class 1, preserving the original TEST class counts. The 2.5th and 97.5th percentiles formed 95% percentile confidence intervals.

Supplementary Method S13. Applicability-domain calculations

Applicability domain (AD) was assessed by two complementary criteria [32,33]. The structural-similarity criterion used Morgan2048 fingerprints for every final model regardless of the winning representation. For each TRAIN molecule, self-similarity was excluded and its nearest-neighbor Tanimoto similarity was recorded. The 5th percentile of this TRAIN leave-one-out nearest-neighbor distribution defined the threshold. A TEST molecule was Tanimoto-AD-IN when its maximum similarity to TRAIN met or exceeded the threshold.

For the leverage criterion, the saved final processed TRAIN and TEST matrices were used. A StandardScaler was fitted to TRAIN only, followed by PCA. The number of components was the smallest number reaching 95% cumulative TRAIN explained variance, capped at 30 components and nTRAIN−2. Leverage was calculated in PCA space after adding an intercept. The warning limit was $h^* = 3(p+1)/\mathrm{nTRAIN}$ [33]. Because of the 30-component cap, the realized explained variance could be below 95%; Supplementary Table S8 reports the actual value. Consensus AD-IN required both Tanimoto-AD-IN and PCA-leverage-AD-IN. No standardized-residual criterion was used because the endpoint was categorical.

Supplementary Method S14. Y-randomization protocol

Y-randomization was applied to the six STRICT primary models and completed to 100 permutations per model [34]. Each permutation preserved the locked feature representation, classifier family, optimized hyperparameters, and original cross-validation family. Fold-specific X preprocessing was repeated according to the frozen training policy, but HPO was not repeated and decision thresholds were not retuned. The randomized statistic was mean CV-MCC; the observed comparator was the CV-MCC of the HPO-selected final configuration. Because the observed configuration had already been selected by HPO whereas the randomized-label runs used fixed hyperparameters, this protocol is not a fully exchangeable permutation test of the entire model-selection pipeline and may favor the observed comparator. It was therefore interpreted as a fixed-configuration chance-correlation stress test.

$$p_{\mathrm{emp}} = [1 + \#(\mathrm{MCCrandom} \geq \mathrm{MCCobserved})] / (\mathrm{Nperm} + 1)$$

With 100 permutations, the minimum attainable empirical p-value is 1/101 = 0.00990. Completed permutation rows were checkpointed and reused across interrupted sessions. The full results appear in Supplementary Table S9 and Supplementary Figure S2.

Supplementary Method S15. SHAP implementation and Morgan-bit mapping

SHAP was applied only to the six STRICT primary models [9]. TreeExplainer was used for ExtraTrees/Random Forest, LinearExplainer for logistic regression, and a computationally capped KernelExplainer for RBF-SVC.

- Tree and linear models: at most 250 TEST molecules were explained.
- Logistic regression: the SHAP background contained up to 200 randomly selected TRAIN molecules.
- RBF-SVC: background = up to 30 TRAIN molecules; explanation set = up to 40 TEST molecules; KernelExplainer nsamples = 120.
- Global importance = mean absolute SHAP; mean signed SHAP was retained to indicate direction toward class 1 (positive) or class 0 (negative).
- Global output retained the highest-ranking features; local explanations preferentially sampled misclassified and high-confidence TEST molecules.
- For Morgan-containing winners, up to the 10 highest-ranking Morgan bits were mapped to representative TRAIN atom environments using RDKit fingerprint bit information. Fragment SMILES and highlighted molecule images were saved.

Because Morgan fingerprints are hashed, the same bit can represent more than one atom environment. Mapped fragments are therefore representative structural realizations rather than unique definitions or causal pharmacophores. Primary-model SHAP summaries and top features are reported in Supplementary Tables S10–S11; complementary CA I-containing global SHAP plots appear in Supplementary Figures S4–S6.

Supplementary Method S16. Reproducibility, integrity, and computational safeguards

- Stage-level manifests recorded dataset definitions, thresholds, file paths, analysis settings, and interface contracts; critical inputs and final estimators were protected by SHA-256 hashes.
- The central feature-store contract required exactly 1,572 unique standardized InChIKeys and complete feature coverage of all 12 final datasets.
- The outer TEST partitions were locked before HPO and were unavailable for split choice, lightweight screening, HPO ranking, or decision-threshold optimization.
- Final model artifacts were independently reloaded with the saved processed TEST matrices. TEST score vectors, OOF-threshold predictions, final labels, and metrics were regenerated and compared with stored outputs.
- All 12 final STRICT/BROAD models passed the reproducibility audit; maximum absolute score differences were on the order of 10^{-16}.
- The principal random seed was 42; deterministic hash-derived seeds were used for repeated trial/permutation operations.
- Software/database provenance is summarized in Supplementary Table S13. The centralized run manifests did not store the Mordred/SHAP package-version strings directly; however, the frozen SEL02 and SEL05 notebooks used unversioned pip installation commands and were executed in August 2026. The stable package releases available at that execution window were mordredcommunity 2.0.7 and SHAP 0.52.0; these reconstructed versions are reported explicitly in Table S13.

Supplementary Tables

Values shown in these tables are derived from the frozen stage outputs used for the manuscript. Machine-readable CSV versions are included in the GitHub-ready repository package. Counts are exact; selected metrics are rounded for readability in this document.

Supplementary Table S1. Target-level data flow for the four human carbonic anhydrase isoenzymes.

Isoenzyme	ChEMBL target ID	Initial Ki activity rows	Binding-quality-filtered rows	Final unique molecules
CA I	CHEMBL261	11,944	5,618	5,117
CA II	CHEMBL205	14,115	6,123	5,524
CA IX	CHEMBL3594	7,374	5,357	4,928
CA XII	CHEMBL3242	5,618	4,419	4,019

Note: The value 5,618 occurs independently as the CA I binding-quality-filtered row count and as the CA XII initial Ki activity-row count. Both values were re-verified against the frozen NB00 retrieval manifests and are correct.

Note. The final target-level molecule counts correspond to the standardized, maximum-one-Rule-of-Five-violation molecule sources used for pairwise discovery.

Supplementary Table S2. Exact pairwise molecule flow under BROAD and STRICT definitions.

Pair	Variant	Common N	Label 0 N	Label 1 N	Binary N	Dual active	Dual weak	Intermediate / mixed
CAI vs CAII	BROAD	4,852	29	808	837	1,745	580	1,690
CAI vs CAII	STRICT	4,684	23	775	798	1,693	547	1,646
CAI vs CAIX	BROAD	3,800	94	838	932	1,127	469	1,272
CAI vs CAIX	STRICT	3,658	89	816	905	1,085	451	1,217
CAI vs CAXII	BROAD	3,057	39	763	802	1,001	233	1,021
CAI vs CAXII	STRICT	2,897	37	724	761	941	223	972
CAII vs CAIX	BROAD	3,939	182	137	319	2,164	372	1,084
CAII vs CAIX	STRICT	3,761	176	124	300	2,063	347	1,051
CAII vs CAXII	BROAD	3,143	58	174	232	1,920	225	766
CAII vs CAXII	STRICT	2,934	55	145	200	1,784	206	744
CAIX vs CAXII	BROAD	3,694	52	109	161	2,349	352	832
CAIX vs CAXII	STRICT	3,595	52	100	152	2,283	344	816

Note. Label 0 denotes selectivity toward the first isoenzyme named in the pair; label 1 denotes selectivity toward the second. Only label 0 and label 1 molecules enter binary classification.

Supplementary Table S3. Final binary-dataset class balance and integrity checks.

Pair	Variant	N	Label 0	Label 1	Minority N	Minority %	Maj./min.	Imbalance	Unique keys	SMILES mismatch	Integrity
CAI vs CAII	BROAD	837	29	808	29	3.5%	27.86	EXTREME	837	0	Yes
CAI vs CAII	STRICT	798	23	775	23	2.9%	33.70	EXTREME	798	0	Yes
CAI vs CAIX	BROAD	932	94	838	94	10.1%	8.91	HIGH	932	0	Yes
CAI vs CAIX	STRICT	905	89	816	89	9.8%	9.17	HIGH	905	0	Yes
CAI vs CAXII	BROAD	802	39	763	39	4.9%	19.56	EXTREME	802	0	Yes
CAI vs CAXII	STRICT	761	37	724	37	4.9%	19.57	EXTREME	761	0	Yes
CAII vs CAIX	BROAD	319	182	137	137	42.9%	1.33	LOW	319	0	Yes
CAII vs CAIX	STRICT	300	176	124	124	41.3%	1.42	LOW	300	0	Yes
CAII vs CAXII	BROAD	232	58	174	58	25.0%	3.00	MODERATE	232	0	Yes
CAII vs CAXII	STRICT	200	55	145	55	27.5%	2.64	MODERATE	200	0	Yes
CAIX vs CAXII	BROAD	161	52	109	52	32.3%	2.10	MODERATE	161	0	Yes
CAIX vs CAXII	STRICT	152	52	100	52	34.2%	1.92	LOW	152	0	Yes

Note. Integrity PASS required unique standardized InChIKeys, valid binary labels, both classes present, complete required fields, exact agreement with the locked 200/800 nM phenotype, and STRICT⊂BROAD consistency.

Supplementary Table S4. TRAIN-only selector scores for all three candidate outer splits across all 12 datasets. Rank 1 is the selected active split; outer TEST performance was not used for selection.

Pair	Variant	Candidate split	Rank	CV-MCC mean	CV-MCC SD	Macro PR-AUC	Balanced acc.	Minority recall
CAII vs CAIX	BROAD	Scaffold-disjoint	1	0.688	0.086	0.947	0.836	0.789
CAII vs CAIX	BROAD	Random stratified	2	0.589	0.066	0.912	0.789	0.709
CAII vs CAIX	BROAD	KS/maximin	3	0.548	0.137	0.873	0.764	0.672
CAII vs CAIX	STRICT	Scaffold-disjoint	1	0.713	0.061	0.946	0.848	0.788
CAII vs CAIX	STRICT	Random stratified	2	0.596	0.039	0.914	0.787	0.687
CAII vs CAIX	STRICT	KS/maximin	3	0.592	0.055	0.879	0.785	0.677
CAII vs CAXII	BROAD	Scaffold-disjoint	1	0.727	0.079	0.940	0.827	0.675
CAII vs CAXII	BROAD	Random stratified	2	0.700	0.038	0.882	0.797	0.608
CAII vs CAXII	BROAD	KS/maximin	3	0.610	0.088	0.839	0.757	0.543
CAII vs CAXII	STRICT	Random stratified	1	0.639	0.051	0.898	0.786	0.616
CAII vs CAXII	STRICT	Scaffold-disjoint	2	0.573	0.091	0.822	0.740	0.524
CAII vs CAXII	STRICT	KS/maximin	3	0.507	0.015	0.845	0.705	0.454
CAIX vs CAXII	BROAD	Random stratified	1	0.586	0.089	0.828	0.798	0.780
CAIX vs CAXII	BROAD	Scaffold-disjoint	2	0.567	0.103	0.855	0.772	0.659
CAIX vs CAXII	BROAD	KS/maximin	3	0.414	0.166	0.748	0.693	0.524
CAIX vs CAXII	STRICT	Random stratified	1	0.645	0.206	0.889	0.825	0.775
CAIX vs CAXII	STRICT	KS/maximin	2	0.474	0.156	0.832	0.723	0.571
CAIX vs CAXII	STRICT	Scaffold-disjoint	3	0.470	0.072	0.815	0.728	0.608
CAI vs CAII	BROAD	Random stratified	1	0.427	0.061	0.689	0.626	0.256
CAI vs CAII	BROAD	KS/maximin	2	0.110	0.206	0.578	0.519	0.042
CAI vs CAII	BROAD	Scaffold-disjoint	3	0.075	0.141	0.605	0.519	0.042
CAI vs CAII	STRICT	Random stratified	1	0.522	0.158	0.791	0.666	0.333

Pair	Variant	Candidate split	Rank	CV-MCC mean	CV-MCC SD	Macro PR-AUC	Balanced acc.	Minority recall
CAI vs CAII	STRICT	Scaffold-disjoint	2	0.336	0.307	0.757	0.610	0.222
CAI vs CAII	STRICT	KS/maximin	3	0.000	0.000	0.626	0.500	0.000
CAI vs CAIX	BROAD	Scaffold-disjoint	1	0.757	0.046	0.924	0.806	0.613
CAI vs CAIX	BROAD	Random stratified	2	0.719	0.182	0.905	0.798	0.600
CAI vs CAIX	BROAD	KS/maximin	3	0.616	0.111	0.870	0.724	0.453
CAI vs CAIX	STRICT	Scaffold-disjoint	1	0.788	0.048	0.930	0.830	0.661
CAI vs CAIX	STRICT	Random stratified	2	0.726	0.070	0.928	0.813	0.635
CAI vs CAIX	STRICT	KS/maximin	3	0.654	0.035	0.869	0.751	0.507
CAI vs CAXII	BROAD	Scaffold-disjoint	1	0.650	0.113	0.862	0.757	0.518
CAI vs CAXII	BROAD	Random stratified	2	0.613	0.057	0.859	0.708	0.418
CAI vs CAXII	BROAD	KS/maximin	3	0.457	0.128	0.776	0.626	0.255
CAI vs CAXII	STRICT	Scaffold-disjoint	1	0.659	0.038	0.839	0.748	0.500
CAI vs CAXII	STRICT	Random stratified	2	0.616	0.136	0.826	0.716	0.433
CAI vs CAXII	STRICT	KS/maximin	3	0.505	0.058	0.806	0.633	0.267

Note: The fixed selector was Morgan2048 + ExtraTreesClassifier with three-fold TRAIN-only cross-validation. Candidate splits were ranked by mean CV-MCC, with macro PR-AUC and balanced accuracy as tie-breakers. No outer TEST performance was evaluated during split selection.

Note. Candidate splits were selected only from cross-validation inside candidate outer TRAIN partitions. “No” in the final column documents that outer TEST performance was not used for active-split selection.

Supplementary Table S5. Full-HPO winning feature/model configuration for each STRICT/BROAD dataset.

Pair	Variant	Role	Outer split	Feature set	Classifier	CV MCC	SD	Macro PR-AUC	Bal. acc.	Minority recall	Trials	TEST used?
CAII vs CAIX	BROAD	SENSITIVITY	Scaffold_disjoint_balanced	RDKit2D_Mordred2D	ExtraTreesClassifier	0.348	0.196	0.754	0.675	0.569	48	No
CAII vs CAIX	STRICT	PRIMARY	Scaffold_disjoint_balanced	RDKit2D	LogisticRegression	0.538	0.112	0.748	0.783	0.840	48	No
CAII vs CAXII	BROAD	SENSITIVITY	Scaffold_disjoint_balanced	Morgan2048	LogisticRegression	0.592	0.261	0.910	0.788	0.641	48	No
CAII vs CAXII	STRICT	PRIMARY	Random_class_balanced	Morgan2048	RandomForestClassifier	0.695	0.072	0.895	0.790	0.589	48	No
CAIX vs CAXII	BROAD	SENSITIVITY	Random_class_balanced	Morgan2048_Mordred2D	ExtraTreesClassifier	0.671	0.068	0.888	0.828	0.758	48	No
CAIX vs CAXII	STRICT	PRIMARY	Random_class_balanced	Morgan2048_RDKit2D_Mordred2D	SVC_RBF	0.667	0.163	0.855	0.829	0.783	48	No
CAI vs CAII	BROAD	SENSITIVITY	Random_class_balanced	Morgan2048	SVC_RBF	0.392	0.376	0.710	0.653	0.310	48	No
CAI vs CAII	STRICT	PRIMARY	Random_class_balanced	Morgan2048_RDKit2D	ExtraTreesClassifier	0.492	0.330	0.750	0.730	0.467	48	No
CAI vs CAIX	BROAD	SENSITIVITY	Scaffold_disjoint_balanced	RDKit2D_Mordred2D	SVC_RBF	0.484	0.177	0.773	0.655	0.314	48	No
CAI vs CAIX	STRICT	PRIMARY	Scaffold_disjoint_balanced	Morgan2048_RDKit2D	LogisticRegression	0.444	0.178	0.719	0.676	0.381	48	No
CAI vs CAXII	BROAD	SENSITIVITY	Scaffold_disjoint_balanced	Morgan2048_Mordred2D	LogisticRegression	0.387	0.285	0.704	0.784	0.640	48	No
CAI vs CAXII	STRICT	PRIMARY	Scaffold_disjoint_balanced	Mordred2D	LogisticRegression	0.422	0.246	0.652	0.788	0.629	48	No

Note. All HPO values were computed on active outer TRAIN only. All 12 winners were frozen in a global lock before final TEST evaluation.

Supplementary Table S6. Final locked outer-TEST performance for all 12 STRICT/BROAD models.

Pair	Variant	Role	Outer split	Feature set	Classifier	TRAIN N	TEST N	CV MCC	OOF threshold	TEST MCC	Macro PR-AUC	Bal. acc.	Macro F1	Minority recall
CAII vs CAIX	BROAD	SENSITIVITY	Scaffold_disjoint_balanced	RDKit2D_Mordred2D	ExtraTreesClassifier	254	65	0.348	0.5073	0.396	0.842	0.687	0.689	0.536
CAII vs CAIX	STRICT	PRIMARY	Scaffold_disjoint_balanced	RDKit2D	LogisticRegression	240	60	0.538	0.5839	0.459	0.768	0.731	0.729	0.720
CAII vs CAXII	BROAD	SENSITIVITY	Scaffold_disjoint_balanced	Morgan2048	LogisticRegression	184	48	0.592	0.4623	-0.031	0.630	0.486	0.482	0.167
CAII vs CAXII	STRICT	PRIMARY	Random_class_balanced	Morgan2048	RandomForestClassifier	160	40	0.695	0.4698	0.748	0.991	0.818	0.857	0.636
CAIX vs CAXII	BROAD	SENSITIVITY	Random_class_balanced	Morgan2048_Mordred2D	ExtraTreesClassifier	128	33	0.671	0.5502	0.614	0.886	0.818	0.804	0.818
CAIX vs CAXII	STRICT	PRIMARY	Random_class_balanced	Morgan2048_RDKit2D_Mordred2D	SVC_RBF	121	31	0.667	0.0133	0.718	0.933	0.859	0.859	0.818
CAI vs CAII	BROAD	SENSITIVITY	Random_class_balanced	Morgan2048	SVC_RBF	669	168	0.392	0.0000	0.402	0.659	0.583	0.635	0.167
CAI vs CAII	STRICT	PRIMARY	Random_class_balanced	Morgan2048_RDKit2D	ExtraTreesClassifier	638	160	0.492	0.6483	0.303	0.624	0.597	0.635	0.200
CAI vs CAIX	BROAD	SENSITIVITY	Scaffold_disjoint_balanced	RDKit2D_Mordred2D	SVC_RBF	744	188	0.484	0.0836	0.518	0.812	0.702	0.746	0.421
CAI vs CAIX	STRICT	PRIMARY	Scaffold_disjoint_balanced	Morgan2048_RDKit2D	LogisticRegression	724	181	0.444	0.3488	0.308	0.638	0.658	0.654	0.389
CAI vs CAXII	BROAD	SENSITIVITY	Scaffold_disjoint_balanced	Morgan2048_Mordred2D	LogisticRegression	641	161	0.387	0.5000	0.289	0.564	0.615	0.639	0.250
CAI vs CAXII	STRICT	PRIMARY	Scaffold_disjoint_balanced	Mordred2D	LogisticRegression	609	152	0.422	0.5000	0.045	0.515	0.530	0.518	0.143

Note. TEST metrics use the OOF-selected threshold. Full SHA-256 final-model hashes are retained in the machine-readable Table S6 CSV and repository FILE_MANIFEST.csv. Main Table 2 reports 95% bootstrap MCC confidence intervals for the six STRICT primary models.

Supplementary Table S7. End-to-end STRICT versus BROAD sensitivity analysis.

Pair	Biological group	STRICT split	BROAD split	STRICT features	BROAD features	STRICT model	BROAD model	STRICT MCC	BROAD MCC	Δ MCC	Same split?	Same features?	Same model?
CAII vs CAIX	Cytosolic–tumor-associated	Scaffold_disjoint_balanced	Scaffold_disjoint_balanced	RDKit2D	RDKit2D_Mordred2D	LogisticRegression	ExtraTreesClassifier	0.459	0.396	-0.063	Yes	No	No
CAII vs CAXII	Cytosolic–tumor-associated	Random_class_balanced	Scaffold_disjoint_balanced	Morgan2048	Morgan2048	RandomForestClassifier	LogisticRegression	0.748	-0.031	-0.779	No	Yes	No
CAIX vs CAXII	Tumor-associated–tumor-associated	Random_class_balanced	Random_class_balanced	Morgan2048_RDKit2D_Mordred2D	Morgan2048_Mordred2D	SVC_RBF	ExtraTreesClassifier	0.718	0.614	-0.104	Yes	No	No
CAI vs CAII	Cytosolic–cytosolic	Random_class_balanced	Random_class_balanced	Morgan2048_RDKit2D	Morgan2048	ExtraTreesClassifier	SVC_RBF	0.303	0.402	0.099	Yes	No	No
CAI vs CAIX	Cytosolic–tumor-associated	Scaffold_disjoint_balanced	Scaffold_disjoint_balanced	Morgan2048_RDKit2D	RDKit2D_Mordred2D	LogisticRegression	SVC_RBF	0.308	0.518	0.210	Yes	No	No
CAI vs CAXII	Cytosolic–tumor-associated	Scaffold_disjoint_balanced	Scaffold_disjoint_balanced	Mordred2D	Morgan2048_Mordred2D	LogisticRegression	LogisticRegression	0.045	0.289	0.244	Yes	No	Yes

Note. These are descriptive end-to-end sensitivity results. Whenever split, feature representation, or model family differs between variants, the MCC difference cannot be attributed solely to the STRICT/BROAD provenance rule. The full machine-readable CSV retains an explicit interpretation-caution field for every pair.

Supplementary Table S8. Applicability-domain diagnostics for all 12 final models.

(A) Structural-similarity domain and consensus coverage.

Pair	Variant	Role	TRAIN N	TEST N	Tanimoto threshold	TEST mean similarity	Tanimoto IN N	Tanimoto IN %	Consensus IN N	Consensus IN %
CAII vs CAIX	BROAD	SENSITIVITY	254	65	0.333	0.480	54	83.1%	54	83.1%
CAII vs CAIX	STRICT	PRIMARY	240	60	0.337	0.471	46	76.7%	45	75.0%
CAII vs CAXII	BROAD	SENSITIVITY	184	48	0.295	0.408	27	56.2%	27	56.2%
CAII vs CAXII	STRICT	PRIMARY	160	40	0.258	0.674	38	95.0%	36	90.0%
CAIX vs CAXII	BROAD	SENSITIVITY	128	33	0.297	0.633	30	90.9%	30	90.9%
CAIX vs CAXII	STRICT	PRIMARY	121	31	0.312	0.618	29	93.5%	29	93.5%
CAI vs CAII	BROAD	SENSITIVITY	669	168	0.383	0.690	159	94.6%	154	91.7%
CAI vs CAII	STRICT	PRIMARY	638	160	0.390	0.700	147	91.9%	138	86.2%
CAI vs CAIX	BROAD	SENSITIVITY	744	188	0.403	0.568	153	81.4%	150	79.8%
CAI vs CAIX	STRICT	PRIMARY	724	181	0.441	0.547	124	68.5%	120	66.3%
CAI vs CAXII	BROAD	SENSITIVITY	641	161	0.411	0.521	110	68.3%	105	65.2%
CAI vs CAXII	STRICT	PRIMARY	609	152	0.396	0.608	137	90.1%	136	89.5%

(B) PCA-leverage domain.

Pair	Variant	PCA components	TRAIN variance explained	h*	PCA IN N	PCA IN %
CAII vs CAIX	BROAD	30	78.0%	0.366	65	100.0%
CAII vs CAIX	STRICT	30	82.5%	0.388	58	96.7%
CAII vs CAXII	BROAD	30	47.8%	0.505	48	100.0%
CAII vs CAXII	STRICT	30	46.2%	0.581	38	95.0%
CAIX vs CAXII	BROAD	30	63.8%	0.727	33	100.0%
CAIX vs CAXII	STRICT	30	66.4%	0.769	31	100.0%
CAI vs CAII	BROAD	30	25.6%	0.139	163	97.0%
CAI vs CAII	STRICT	30	28.6%	0.146	151	94.4%
CAI vs CAIX	BROAD	30	72.4%	0.125	184	97.9%
CAI vs CAIX	STRICT	30	28.2%	0.128	177	97.8%
CAI vs CAXII	BROAD	30	38.1%	0.145	156	96.9%
CAI vs CAXII	STRICT	30	76.6%	0.153	151	99.3%

Note. PCA was capped at 30 components; therefore the actual explained variance can be below the nominal 95% target in high-dimensional feature spaces. Consensus AD-IN requires both Tanimoto and PCA-leverage criteria.

Supplementary Table S9. Y-randomization results for the six STRICT primary models (100 permutations each).

Pair	N perm.	Observed CV MCC	Random mean	Random SD	Random max	Observed − max	Empirical p	Re-HPO?	Retune threshold?
CAII vs CAIX	100	0.538	0.005	0.083	0.184	0.354	0.0099	No	No
CAII vs CAXII	100	0.695	0.010	0.098	0.241	0.453	0.0099	No	No
CAIX vs CAXII	100	0.667	-0.006	0.117	0.239	0.428	0.0099	No	No
CAI vs CAII	100	0.492	-0.001	0.034	0.200	0.292	0.0099	No	No
CAI vs CAIX	100	0.444	-0.002	0.039	0.103	0.341	0.0099	No	No
CAI vs CAXII	100	0.422	0.000	0.000	0.000	0.422	0.0099	No	No

Note: The observed value is the CV-MCC of the HPO-selected final configuration, whereas randomized-label runs used the already selected feature/model/hyperparameter configuration without re-HPO or threshold retuning. The comparison therefore may favor the observed model and is interpreted as a fixed-configuration stress test rather than a fully exchangeable permutation test of the complete model-selection pipeline. For CA I/CA XII, all 100 randomized runs had MCC = 0.000 and macro PR-AUC = 0.500. Together with the strongly regularized L1 logistic configuration (C = 0.005), this pattern is consistent with degenerate constant-score/single-class predictions under randomized labels and is not interpreted as unusually strong biological evidence.

Note. No randomized mean CV-MCC equaled or exceeded the observed locked CV-MCC. With 100 permutations, all six empirical p-values were 1/101 = 0.00990. This rejects chance label association as an explanation for the development/CV signal, but does not establish outer-test generalizability.

Supplementary Table S10. SHAP implementation summary for the six STRICT primary models.

Pair	Feature set	Classifier	Explainer	TEST explained	Processed features	Top global feature	Top mean |SHAP|	Morgan bits mapped
CAII vs CAIX	RDKit2D	LogisticRegression	LinearExplainer	60	162	RDKit2D__SMR_VSA4	0.8816	0
CAII vs CAXII	Morgan2048	RandomForestClassifier	TreeExplainer	40	1,042	Morgan2048_bit_1452	0.0206	10
CAIX vs CAXII	Morgan2048_RDKit2D_Mordred2D	SVC_RBF	KernelExplainer	31	1,629	Morgan2048_bit_1440	0.0112	10
CAI vs CAII	Morgan2048_RDKit2D	ExtraTreesClassifier	TreeExplainer	160	1,764	Morgan2048_bit_935	0.0160	10
CAI vs CAIX	Morgan2048_RDKit2D	LogisticRegression	LinearExplainer	181	1,797	RDKit2D__fr_bicyclic	0.6592	10
CAI vs CAXII	Mordred2D	LogisticRegression	LinearExplainer	152	697	Mordred2D__BCUTd-1h	0.1389	0

Note. Positive signed SHAP contributions favor class 1 (the second isoenzyme named in the pair), whereas negative values favor class 0.

Supplementary Table S11. Ten highest-ranked global SHAP features for each STRICT primary model.

Pair	Rank	Feature	Mean |SHAP|	Mean signed SHAP	Representative mapped Morgan fragment
CAI vs CAII	1	Morgan2048_bit_935	0.0160	0.0063	N
CAI vs CAII	2	Morgan2048_bit_1607	0.0094	0.0070	NS
CAI vs CAII	3	Morgan2048_bit_1171	0.0089	0.0069	N
CAI vs CAII	4	Morgan2048_bit_189	0.0089	0.0089	cC#Cc(c)c
CAI vs CAII	5	Morgan2048_bit_430	0.0088	0.0074	C#Cc
CAI vs CAII	6	Morgan2048_bit_378	0.0083	0.0036	n
CAI vs CAII	7	Morgan2048_bit_833	0.0082	0.0054	cS(N)(=O)=O
CAI vs CAII	8	Morgan2048_bit_1734	0.0075	0.0075	Cc(c)ccc
CAI vs CAII	9	Morgan2048_bit_1476	0.0075	0.0065	O=S
CAI vs CAII	10	Morgan2048_bit_102	0.0062	0.0050	Cc(c)c
CAI vs CAIX	1	RDKit2D__fr_bicyclic	0.6592	-0.1765	
CAI vs CAIX	2	RDKit2D__SMR_VSA3	0.6355	-0.2176	
CAI vs CAIX	3	RDKit2D__PEOE_VSA10	0.5199	0.1299	
CAI vs CAIX	4	RDKit2D__BCUT2D_MWLOW	0.5153	-0.0585	
CAI vs CAIX	5	RDKit2D__PEOE_VSA7	0.4703	0.0034	
CAI vs CAIX	6	RDKit2D__SMR_VSA10	0.4604	0.0074	
CAI vs CAIX	7	RDKit2D__MinEStateIndex	0.4550	-0.2749	
CAI vs CAIX	8	RDKit2D__BCUT2D_CHGLO	0.4489	0.0358	
CAI vs CAIX	9	RDKit2D__fr_piperzine	0.4340	0.0530	
CAI vs CAIX	10	RDKit2D__fr_prisulfonamd	0.4316	0.0179	
CAI vs CAXII	1	Mordred2D__BCUTd-1h	0.1389	0.0815	
CAI vs CAXII	2	Mordred2D__Xch-3d	0.0379	-0.0067	
CAI vs CAXII	3	Mordred2D__MAXssCH2	0.0000	0.0000	
CAI vs CAXII	4	Mordred2D__MAXtCH	0.0000	0.0000	
CAI vs CAXII	5	Mordred2D__MAXdsCH	0.0000	0.0000	
CAI vs CAXII	6	Mordred2D__MAXaaCH	0.0000	0.0000	
CAI vs CAXII	7	Mordred2D__MAXsssCH	0.0000	0.0000	
CAI vs CAXII	8	Mordred2D__MAXtsC	0.0000	0.0000	
CAI vs CAXII	9	Mordred2D__MAXdssC	0.0000	0.0000	
CAI vs CAXII	10	Mordred2D__MAXaasC	0.0000	0.0000	

Pair	Rank	Feature	Mean |SHAP|	Mean signed SHAP	Representative mapped Morgan fragment
CAII vs CAIX	1	RDKit2D__SMR_VSA4	0.8816	0.2487	
CAII vs CAIX	2	RDKit2D__PEOE_VSA3	0.8207	0.1382	
CAII vs CAIX	3	RDKit2D__PEOE_VSA4	0.8049	-0.1310	
CAII vs CAIX	4	RDKit2D__fr_ketone	0.8015	-0.2323	
CAII vs CAIX	5	RDKit2D__NumHAcceptors	0.7415	0.5206	
CAII vs CAIX	6	RDKit2D__SMR_VSA10	0.7248	0.1874	
CAII vs CAIX	7	RDKit2D__fr_nitro	0.6174	-0.2987	
CAII vs CAIX	8	RDKit2D__VSA_EState2	0.6025	-0.2817	
CAII vs CAIX	9	RDKit2D__fr_aniline	0.5635	0.1585	
CAII vs CAIX	10	RDKit2D__fr_prisulfonamd	0.5481	-0.0050	
CAII vs CAXII	1	Morgan2048_bit_1452	0.0206	0.0127	cnc
CAII vs CAXII	2	Morgan2048_bit_1750	0.0120	-0.0004	ccc
CAII vs CAXII	3	Morgan2048_bit_1602	0.0119	0.0042	cO
CAII vs CAXII	4	Morgan2048_bit_310	0.0118	0.0034	cccc(C)c
CAII vs CAXII	5	Morgan2048_bit_191	0.0116	0.0044	cc(N)c
CAII vs CAXII	6	Morgan2048_bit_1970	0.0115	0.0011	cc(O)c
CAII vs CAXII	7	Morgan2048_bit_926	0.0113	0.0039	C
CAII vs CAXII	8	Morgan2048_bit_80	0.0102	0.0023	C
CAII vs CAXII	9	Morgan2048_bit_1019	0.0079	0.0034	C
CAII vs CAXII	10	Morgan2048_bit_1536	0.0072	-0.0000	cOC
CAIX vs CAXII	1	Morgan2048_bit_1440	0.0112	-0.0050	cc(C)cc(c)c
CAIX vs CAXII	2	RDKit2D__EState_VSA10	0.0094	0.0018	
CAIX vs CAXII	3	Morgan2048_bit_86	0.0091	-0.0091	Cc(n)cn(C)n
CAIX vs CAXII	4	Mordred2D__ATSC1i	0.0090	-0.0090	
CAIX vs CAXII	5	Mordred2D__AATSC8p	0.0088	-0.0088	
CAIX vs CAXII	6	Mordred2D__NaasN	0.0085	-0.0085	
CAIX vs CAXII	7	Mordred2D__BCUTc-1l	0.0083	-0.0083	
CAIX vs CAXII	8	Morgan2048_bit_1670	0.0082	-0.0082	cc(F)c
CAIX vs CAXII	9	Mordred2D__AATS3d	0.0082	-0.0004	
CAIX vs CAXII	10	Morgan2048_bit_1528	0.0081	-0.0081	cc(N)c(c(c)S)C(=O)N

Note. The fragment column is populated only when the ranked feature is a Morgan bit and a representative TRAIN atom environment was successfully mapped. Hashed-bit mappings are representative, not unique, structural realizations.

Supplementary Table S12. Independent artifact-reproducibility audit for all 12 final models.

Pair	Variant	Role	Classifier	Feature set	TEST N	Max |score diff|	Predictions	Threshold	Metrics	PASS
CAII vs CAIX	BROAD	SENSITIVITY	ExtraTreesClassifier	RDKit2D_Mordred2D	65	1.67e-16	Yes	Yes	Yes	Yes
CAII vs CAIX	STRICT	PRIMARY	LogisticRegression	RDKit2D	60	1.11e-16	Yes	Yes	Yes	Yes
CAII vs CAXII	BROAD	SENSITIVITY	LogisticRegression	Morgan2048	48	1.11e-16	Yes	Yes	Yes	Yes
CAII vs CAXII	STRICT	PRIMARY	RandomForestClassifier	Morgan2048	40	0.00e+00	Yes	Yes	Yes	Yes
CAIX vs CAXII	BROAD	SENSITIVITY	ExtraTreesClassifier	Morgan2048_Mordred2D	33	1.11e-16	Yes	Yes	Yes	Yes
CAIX vs CAXII	STRICT	PRIMARY	SVC_RBF	Morgan2048_RDKit2D_Mordred2D	31	2.22e-16	Yes	Yes	Yes	Yes
CAI vs CAII	BROAD	SENSITIVITY	SVC_RBF	Morgan2048	168	4.44e-16	Yes	Yes	Yes	Yes
CAI vs CAII	STRICT	PRIMARY	ExtraTreesClassifier	Morgan2048_RDKit2D	160	2.22e-16	Yes	Yes	Yes	Yes
CAI vs CAIX	BROAD	SENSITIVITY	SVC_RBF	RDKit2D_Mordred2D	188	2.22e-16	Yes	Yes	Yes	Yes
CAI vs CAIX	STRICT	PRIMARY	LogisticRegression	Morgan2048_RDKit2D	181	1.11e-16	Yes	Yes	Yes	Yes
CAI vs CAXII	BROAD	SENSITIVITY	LogisticRegression	Morgan2048_Mordred2D	161	5.55e-17	Yes	Yes	Yes	Yes
CAI vs CAXII	STRICT	PRIMARY	LogisticRegression	Mordred2D	152	5.55e-17	Yes	Yes	Yes	Yes

Note. Reproducibility was assessed after reloading the saved estimator and saved processed TEST matrix. All stored score vectors, OOF-derived threshold predictions, final labels, and metrics were recovered within numerical tolerance.

Supplementary Table S13. Publication-run software and database provenance recorded by the frozen workflow.

Component	Version / release	Evidence recorded in frozen workflow	Role
ChEMBL database	Release 37	Release date 1 May 2026; retrieval 18 August 2026	Experimental Ki bioactivity and assay/document provenance
Python	3.12.13	Database/standardization manifests	Workflow runtime
pandas	2.2.3	Database/standardization manifests	Tabular processing
NumPy	2.0.2	Database/standardization manifests	Numerical processing
RDKit	2026.03.5	Frozen workflow manifests	Standardization, descriptors, fingerprints, scaffolds
Matplotlib	3.10.0	Database/standardization manifests	Plot generation
scikit-learn	1.6.1	Frozen estimator environment	Splitting, preprocessing, models, validation
Mordred-compatible descriptors	mordredcommunity 2.0.7	Version reconstructed from frozen SEL02 unpinned pip installation and August 2026 execution date	2D descriptor generation
SHAP	0.52.0	Version reconstructed from frozen SEL05 unpinned pip installation and August 2026 execution date	Feature attribution
Principal random seed	42	Project-wide setting	Deterministic modeling and validation

Note: mordredcommunity 2.0.7 (released 22 January 2026) and SHAP 0.52.0 (released 28 May 2026) were the stable package releases available when the frozen SEL02/SEL05 notebooks executed their unpinned pip installations in August 2026.

Note. Exact package versions are reported only where they were captured by the frozen manifests/environment records. The Supplementary Material does not infer unrecorded versions from the present execution environment.

Supplementary Figures

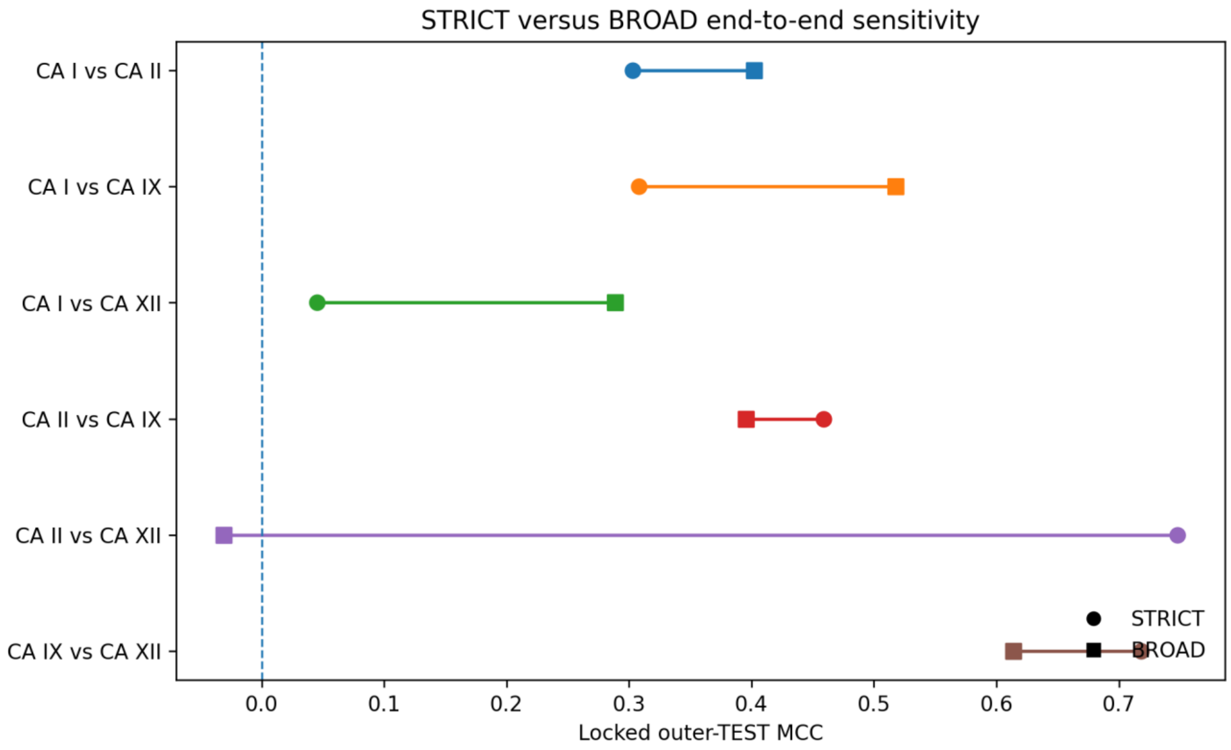

Supplementary Figure S1. STRICT versus BROAD end-to-end sensitivity in locked outer-TEST MCC. Circles denote STRICT; squares denote BROAD. Lines connect the two independently optimized analysis variants for each isoenzyme pair. These differences are descriptive sensitivity results rather than isolated provenance effects.

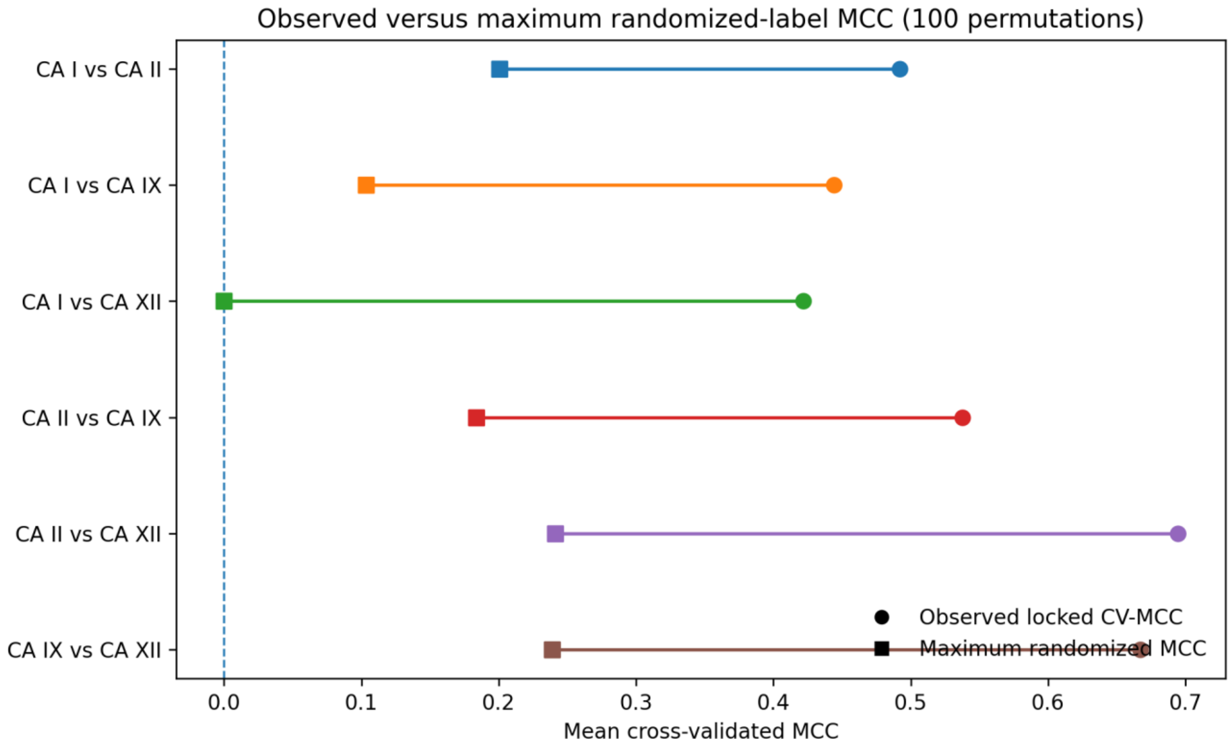

Supplementary Figure S2. Observed locked cross-validated MCC versus the maximum MCC from 100 randomized-label permutations for each STRICT primary model. Circles denote observed CV-MCC; squares denote the maximum randomized mean CV-MCC. All empirical p-values were 1/101 = 0.00990.

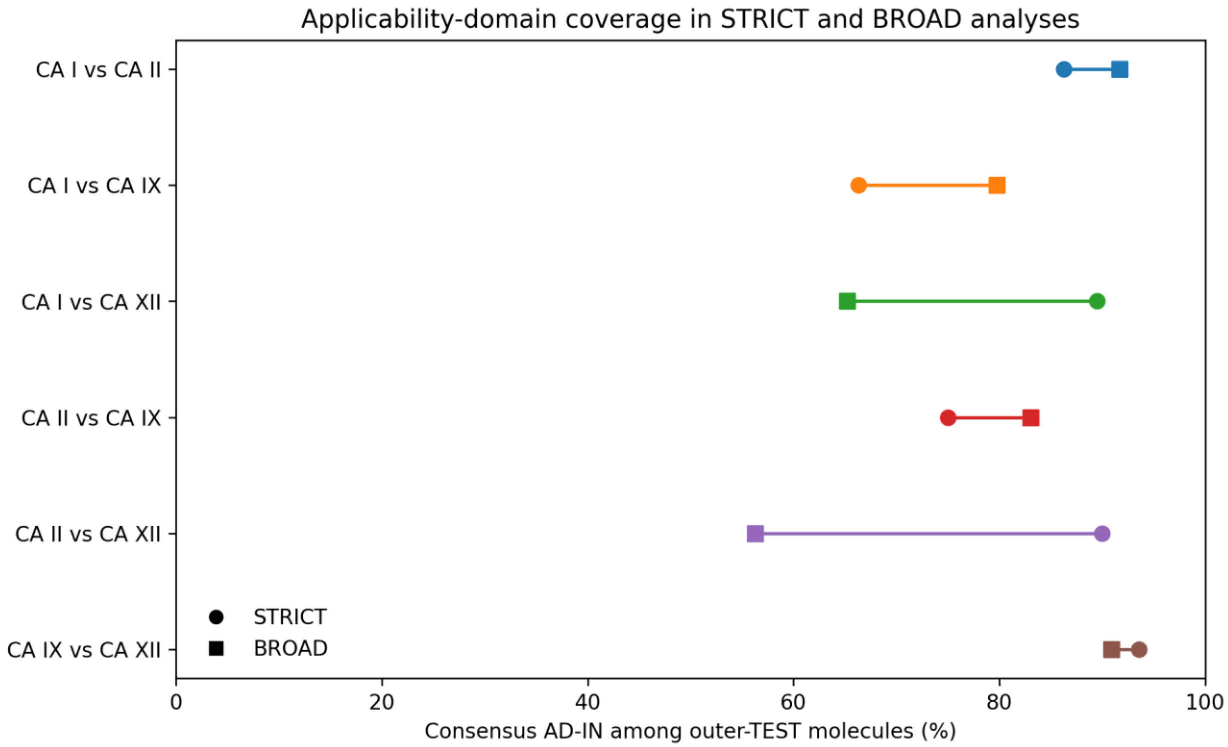

Supplementary Figure S3. Consensus applicability-domain coverage in STRICT and BROAD analyses. Circles denote STRICT; squares denote BROAD. Consensus AD-IN requires both the Morgan/Tanimoto structural-similarity criterion and the PCA-leverage criterion.

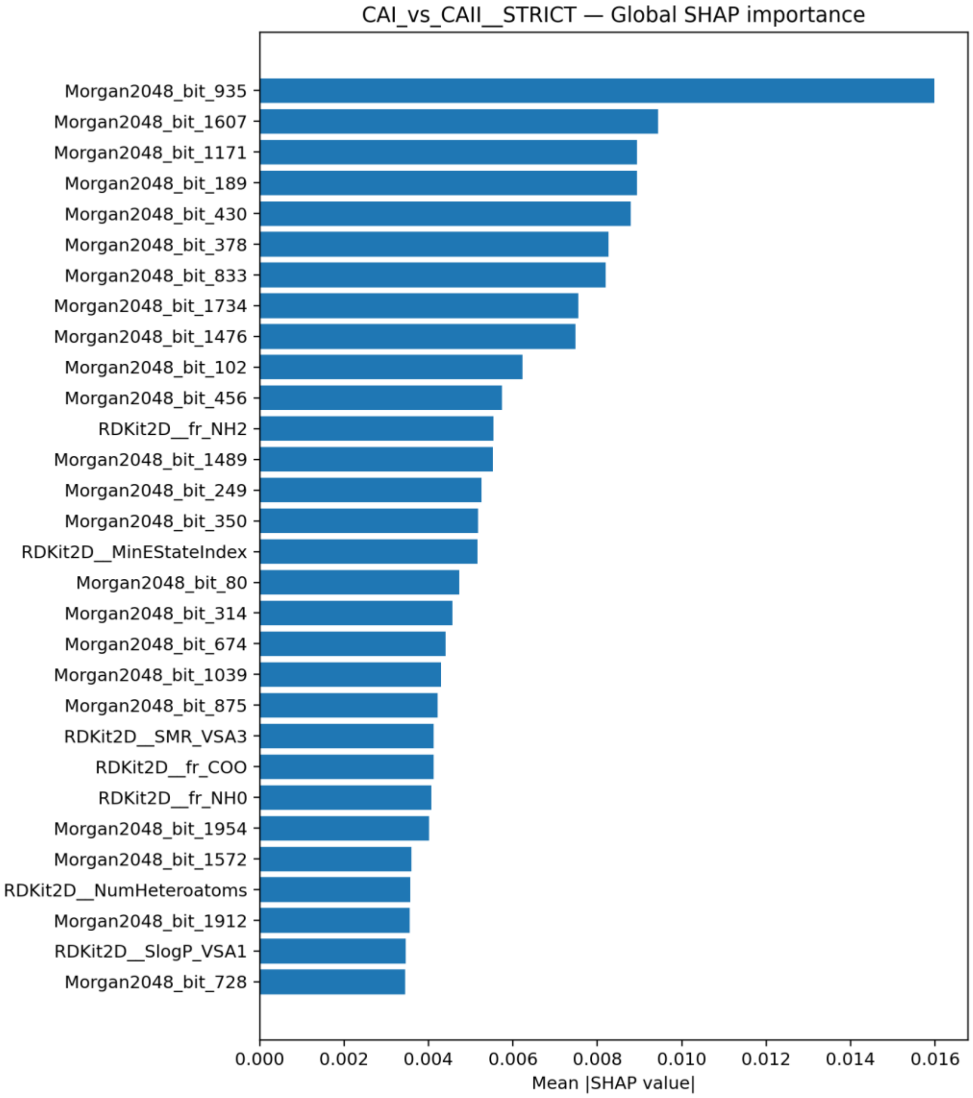

Supplementary Figure S4. Global SHAP importance for the primary STRICT CA I vs CA II model (ExtraTrees; Morgan2048+RDKit2D). The model had outer-TEST MCC = 0.303. Mean absolute SHAP values quantify global contribution magnitude, not causal importance.

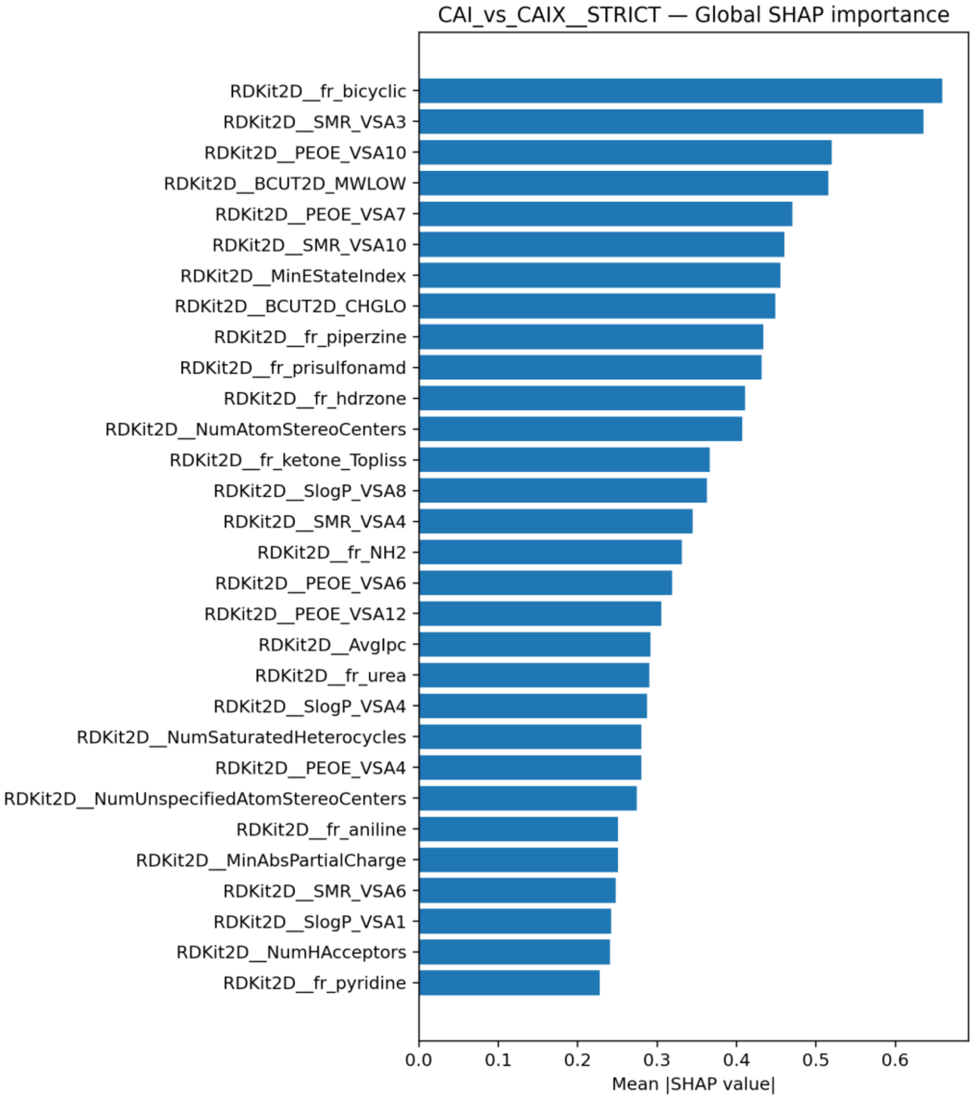

Supplementary Figure S5. Global SHAP importance for the primary STRICT CA I vs CA IX model (logistic regression; Morgan2048+RDKit2D). The leading global feature was RDKit2D__fr_bicyclic.

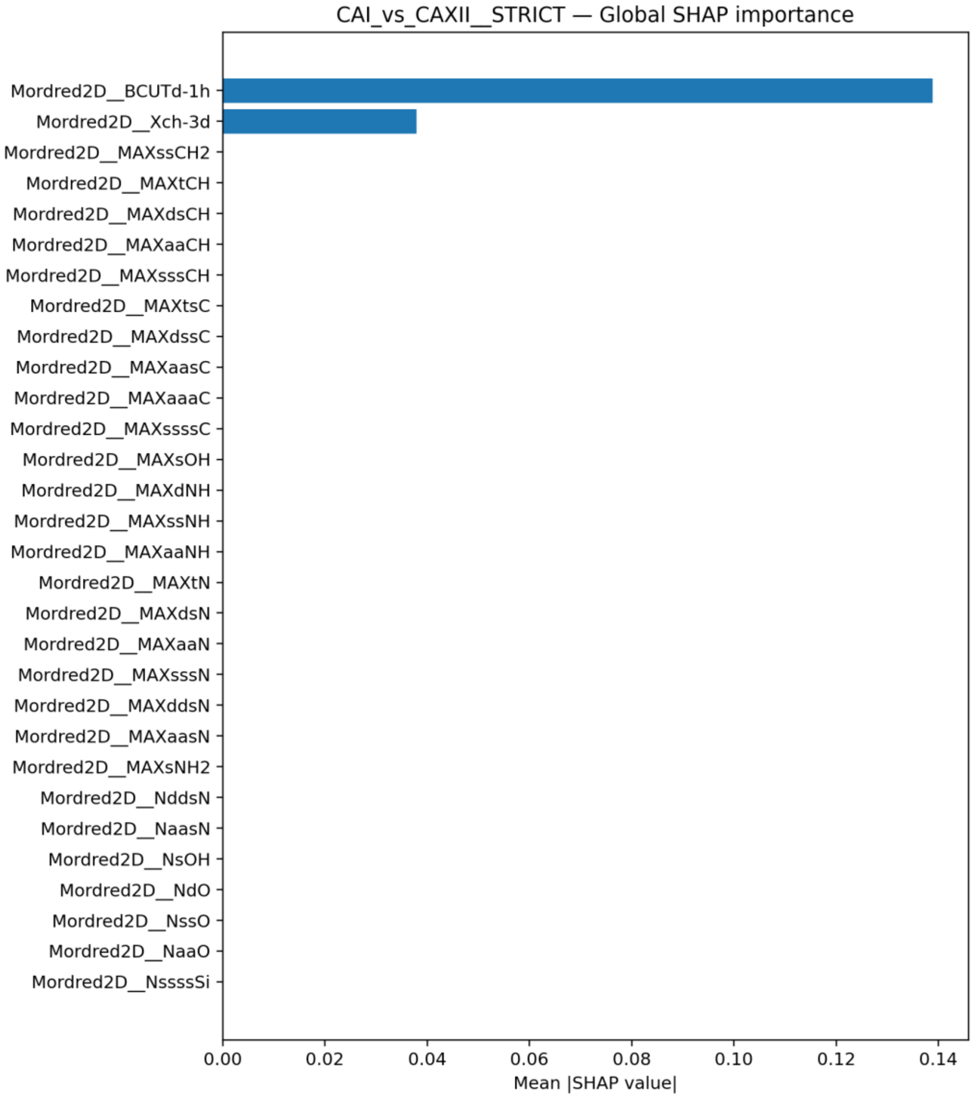

Supplementary Figure S6. Global SHAP importance for the primary STRICT CA I vs CA XII model (logistic regression; Mordred2D). The leading feature was Mordred2D__BCUTd-1h. This model generalized poorly on the locked outer TEST despite high consensus AD coverage.

Supplementary Post-hoc Sensitivity Analyses

The analyses in this section were performed post hoc after the primary STRICT/BROAD splits, model families, hyperparameters, OOF thresholds, and outer-TEST predictions had been frozen. No primary model was re-selected. The purpose was to characterize threshold dependence, minority-class support, applicability-domain subgroup behavior, and residual experimental heterogeneity without altering the frozen primary modeling decisions.

Supplementary Table S14. STRICT cohort size and class composition under alternative operational Ki thresholds. The primary 200/800 nM definition imposes a minimum fourfold potency separation; alternative scenarios were evaluated post hoc and were not used to re-select the primary models.

Pair	Threshold scenario	Minimum fold separation	Class 0 N	Class 1 N	Binary N
CA I vs CA II	100_1000	10.000	14	507	521
CA I vs CA II	100_800	8.000	16	649	665
CA I vs CA II	200_1000	5.000	19	617	636
CA I vs CA II	200_800_PRIMARY	4.000	23	775	798
CA I vs CA II	300_600	2.000	44	1094	1138
CA I vs CA IX	100_1000	10.000	64	572	636
CA I vs CA IX	100_800	8.000	80	695	775
CA I vs CA IX	200_1000	5.000	73	677	750
CA I vs CA IX	200_800_PRIMARY	4.000	89	816	905
CA I vs CA IX	300_600	2.000	132	1064	1196
CA I vs CA XII	100_1000	10.000	22	566	588
CA I vs CA XII	100_800	8.000	33	673	706
CA I vs CA XII	200_1000	5.000	26	609	635
CA I vs CA XII	200_800_PRIMARY	4.000	37	724	761
CA I vs CA XII	300_600	2.000	53	909	962
CA II vs CA IX	100_1000	10.000	138	74	212
CA II vs CA IX	100_800	8.000	160	104	264
CA II vs CA IX	200_1000	5.000	152	88	240
CA II vs CA IX	200_800_PRIMARY	4.000	176	124	300
CA II vs CA IX	300_600	2.000	232	231	463
CA II vs CA XII	100_1000	10.000	35	95	130
CA II vs CA XII	100_800	8.000	54	127	181
CA II vs CA XII	200_1000	5.000	35	110	145
CA II vs CA XII	200_800_PRIMARY	4.000	55	145	200
CA II vs CA XII	300_600	2.000	95	209	304
CA IX vs CA XII	100_1000	10.000	16	63	79
CA IX vs CA XII	100_800	8.000	35	85	120
CA IX vs CA XII	200_1000	5.000	23	74	97
CA IX vs CA XII	200_800_PRIMARY	4.000	52	100	152
CA IX vs CA XII	300_600	2.000	109	145	254

Supplementary Table S15. Performance of the originally locked primary TEST predictions within nested stricter threshold subsets. No retraining, repartitioning, HPO, or threshold retuning was performed; results are descriptive and increasingly uncertain as subgroup size decreases.

Pair	Scenario	N	Class 0	Class 1	MCC	Balanced accuracy	Minority recall
CA I vs CA II	100_1000	105	3	102	0.000	0.500	0.000
CA I vs CA II	100_800	133	3	130	-0.013	0.496	0.000
CA I vs CA II	200_1000	128	4	124	0.494	0.625	0.250
CA I vs CA II	200_800_PRIMARY	160	5	155	0.303	0.597	0.200
CA I vs CA IX	100_1000	113	14	99	0.450	0.694	0.429
CA I vs CA IX	100_800	145	18	127	0.373	0.667	0.389
CA I vs CA IX	200_1000	147	14	133	0.350	0.680	0.429
CA I vs CA IX	200_800_PRIMARY	181	18	163	0.308	0.658	0.389
CA I vs CA XII	100_1000	114	2	112	0.225	0.719	0.500
CA I vs CA XII	100_800	137	5	132	0.106	0.570	0.200
CA I vs CA XII	200_1000	127	4	123	0.105	0.584	0.250

CA I vs CA XII	200_800_PRIMARY	152	7	145	0.045	0.530	0.143
CA II vs CA IX	100_1000	42	29	13	0.633	0.841	0.923
CA II vs CA IX	100_800	51	30	21	0.523	0.764	0.762
CA II vs CA IX	200_1000	48	34	14	0.543	0.796	0.857
CA II vs CA IX	200_800_PRIMARY	60	35	25	0.459	0.731	0.720
CA II vs CA XII	100_1000	25	8	17	0.729	0.812	0.625
CA II vs CA XII	100_800	33	11	22	0.734	0.818	0.636
CA II vs CA XII	200_1000	32	8	24	0.745	0.812	0.625
CA II vs CA XII	200_800_PRIMARY	40	11	29	0.748	0.818	0.636
CA IX vs CA XII	100_1000	12	3	9	0.816	0.944	1.000
CA IX vs CA XII	100_800	24	8	16	0.625	0.812	0.750
CA IX vs CA XII	200_1000	16	4	12	0.856	0.958	1.000
CA IX vs CA XII	200_800_PRIMARY	31	11	20	0.718	0.859	0.818

Supplementary Table S16. Model-default and OOF-selected operating thresholds for the six primary STRICT models. The RBF-SVC uses a decision-function default cutoff of 0; probability-like models use 0.5. OOF tuning was completed before outer-TEST evaluation.

Pair	Default cutoff	OOF cutoff	OOF N	OOF MCC default	OOF MCC tuned	Locked TEST MCC
CA I vs CA II	0.500	0.648	638	0.436	0.527	0.303
CA I vs CA IX	0.500	0.349	724	0.390	0.408	0.308
CA I vs CA XII	0.500	0.500	609	0.553	0.553	0.045
CA II vs CA IX	0.500	0.584	240	0.507	0.509	0.459
CA II vs CA XII	0.500	0.470	160	0.657	0.677	0.748
CA IX vs CA XII	0.000	0.013	121	0.652	0.672	0.718

Supplementary Table S17. Minority-class representation in the six primary STRICT datasets and their locked outer TRAIN/TEST partitions.

Pair	Total N	Minority N	Maj:min ratio	TRAIN c0	TRAIN c1	TEST c0	TEST c1
CA I vs CA II	798	23	33.696	18	620	5	155
CA I vs CA IX	905	89	9.169	71	653	18	163
CA I vs CA XII	761	37	19.568	30	579	7	145
CA II vs CA IX	300	124	1.419	141	99	35	25
CA II vs CA XII	200	55	2.636	44	116	11	29
CA IX vs CA XII	152	52	1.923	41	80	11	20

Supplementary Table S18. Descriptive performance of the frozen primary STRICT TEST predictions stratified by consensus applicability-domain status. Metrics from very small or single-class-support subgroups should not be interpreted as stable discrimination estimates.

Pair	AD subgroup	N	Class 0	Class 1	MCC	Balanced accuracy	Minority recall	Status
CA I vs CA II	AD-IN	138	2	136	0.000	0.500	0.000	descriptive
CA I vs CA II	AD-OUT	22	3	19	0.335	0.640	0.333	descriptive
CA I vs CA IX	AD-IN	120	9	111	0.520	0.760	0.556	descriptive
CA I vs CA IX	AD-OUT	61	9	52	0.066	0.534	0.222	descriptive
CA I vs CA XII	AD-IN	136	5	131	-0.046	0.473	0.000	descriptive
CA I vs CA XII	AD-OUT	16	2	14	0.098	0.571	0.500	descriptive
CA II vs CA IX	AD-IN	45	25	20	0.460	0.730	0.700	descriptive
CA II vs CA IX	AD-OUT	15	10	5	0.472	0.750	0.800	descriptive
CA II vs CA XII	AD-IN	36	9	27	0.775	0.833	0.667	descriptive
CA II vs CA XII	AD-OUT	4	2	2	0.577	0.750	1.000	very small subgroup; metrics unstable
CA IX vs CA XII	AD-IN	29	10	19	0.695	0.847	0.800	descriptive
CA IX vs CA XII	AD-OUT	2	1	1	1.000	1.000	1.000	insufficient class support; discrimination metrics not interpretable

Supplementary Table S19. Residual molecule-level activity heterogeneity within the frozen STRICT datasets. Low duplicate measurement/assay counts do not eliminate unrecorded or incompletely standardized protocol-level differences across experiments.

Pair	Isoenzyme	N	Multiple measurements N	Multiple assays N	pKi SD available N	Median pKi SD	Max pKi SD	High-variability N
CA I vs CA II	CA I	798	3	1	3	0.116	0.362	0
CA I vs CA II	CA II	798	3	1	3	0.149	0.156	0
CA I vs CA IX	CA I	905	5	3	5	0.062	0.362	0
CA I vs CA IX	CA IX	905	3	1	3	0.005	0.024	0
CA I vs CA XII	CA I	761	5	3	5	0.062	0.362	0
CA I vs CA XII	CA XII	761	3	1	3	0.003	0.353	0
CA II vs CA IX	CA II	300	1	1	1	0.018	0.018	0
CA II vs CA IX	CA IX	300	0	0	0	—	—	0
CA II vs CA XII	CA II	200	1	1	1	0.018	0.018	0
CA II vs CA XII	CA XII	200	0	0	0	—	—	0
CA IX vs CA XII	CA IX	152	0	0	0	—	—	0
CA IX vs CA XII	CA XII	152	0	0	0	—	—	0

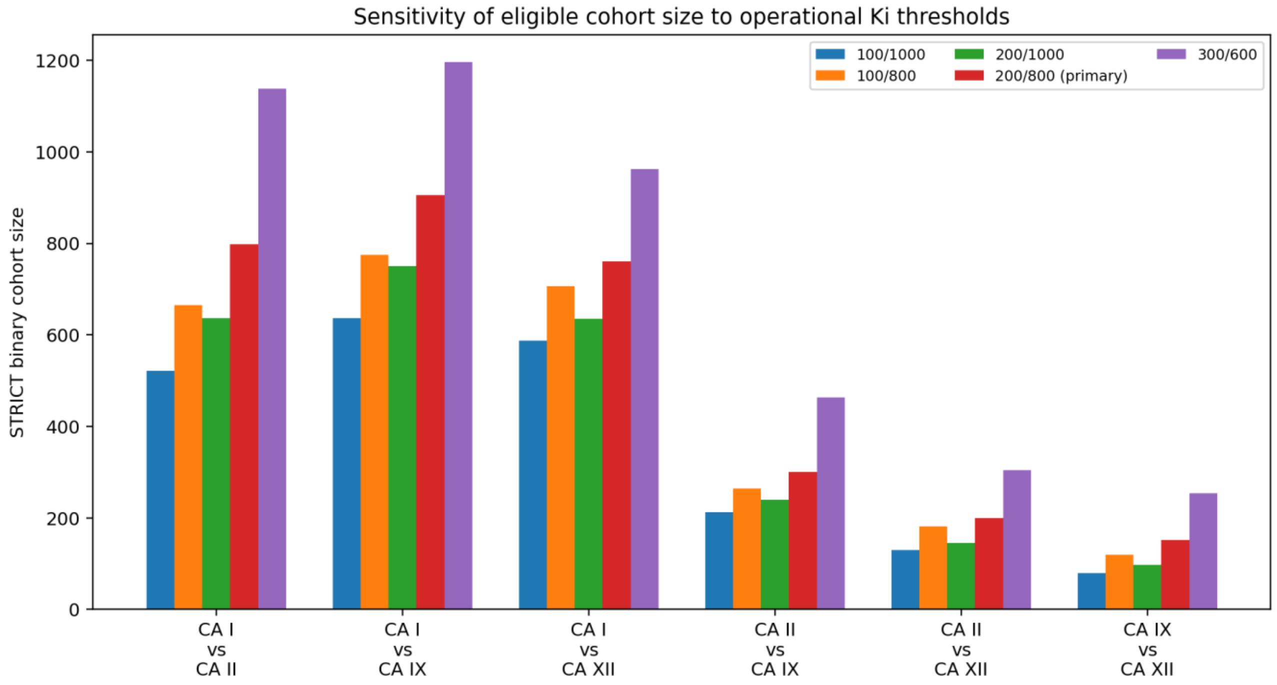

Supplementary Figure S7. Effect of alternative operational Ki thresholds on the number of STRICT binary molecules available for each pairwise task. More stringent thresholds reduce cohort size and often reduce minority support.

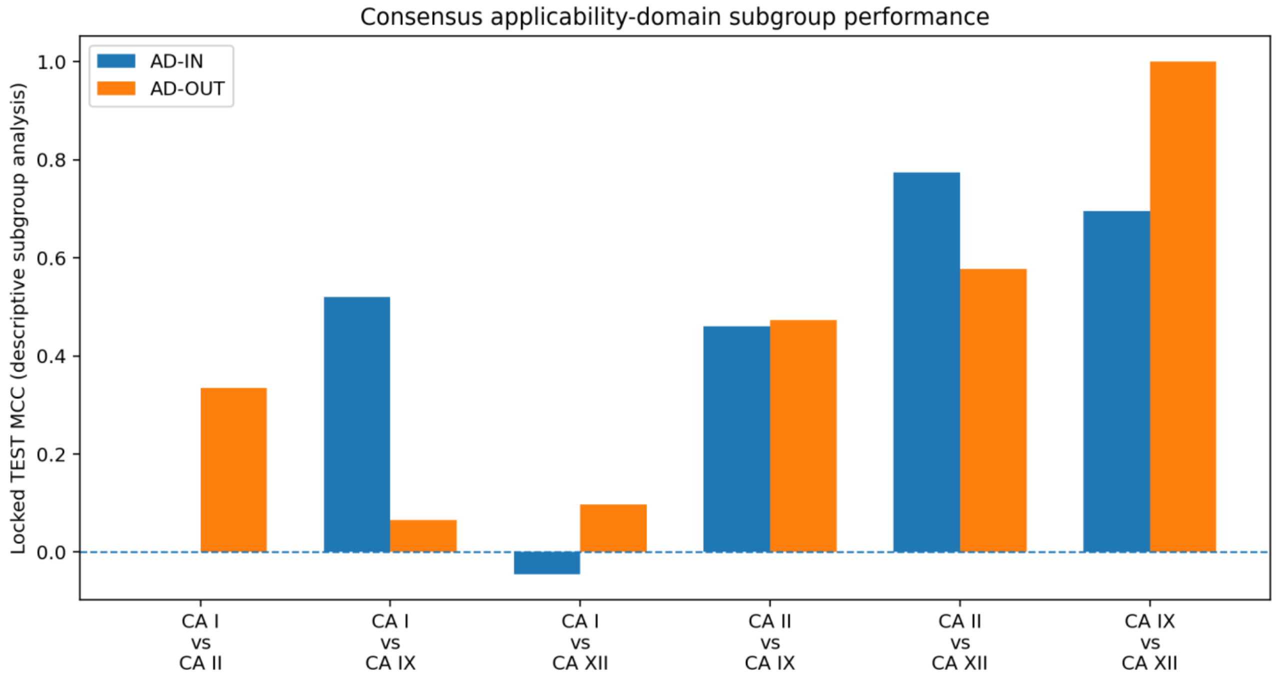

Supplementary Figure S8. Descriptive MCC of the frozen primary STRICT TEST predictions within consensus AD-IN and AD-OUT subgroups. Subgroups with very small N are shown for completeness but are not interpreted as stable discrimination estimates.

References cited in the Supplementary Methods

[1] Landrum G. RDKit: Open-source cheminformatics. Release 2014.03.1. Zenodo. 2014. doi:10.5281/zenodo.10398.

[2] Bento AP, Hersey A, Félix E, Landrum G, Gaulton A, Atkinson F, et al. An open source chemical structure curation pipeline using RDKit. Journal of Cheminformatics. 2020;12. doi:10.1186/s13321-020-00456-1.

[3] Moriwaki H, Tian Y-S, Kawashita N, Takagi T. Mordred: a molecular descriptor calculator. Journal of Cheminformatics. 2018;10:4. doi:10.1186/s13321-018-0258-y.

[4] Rogers D, Hahn M. Extended-Connectivity Fingerprints. Journal of Chemical Information and Modeling. 2010;50:742–754. doi:10.1021/ci100050t.

[5] Pedregosa F, Varoquaux G, Gramfort A, Michel V, Thirion B, Grisel O, et al. Scikit-learn: Machine Learning in Python. Journal of Machine Learning Research. 2011;12:2825–2830.

[6] Cortes C, Vapnik V. Support-Vector Networks. Machine Learning. 1995;20:273–297. doi:10.1007/BF00994018.

[7] Chicco D, Jurman G. The advantages of the Matthews correlation coefficient (MCC) over F1 score and accuracy in binary classification evaluation. BMC Genomics. 2020;21:6. doi:10.1186/s12864-019-6413-7.

[8] Saito T, Rehmsmeier M. The Precision-Recall Plot Is More Informative than the ROC Plot When Evaluating Binary Classifiers on Imbalanced Datasets. PLOS ONE. 2015;10:e0118432. doi:10.1371/journal.pone.0118432.

[9] Lundberg SM, Lee S-I. A Unified Approach to Interpreting Model Predictions. Advances in Neural Information Processing Systems 30. 2017:4765–4774.

[28] Zdrazil B, Felix E, Hunter F, Manners EJ, Blackshaw JA, Corbett S, et al. The ChEMBL Database in 2023: a drug discovery platform spanning multiple bioactivity data types and time periods. Nucleic Acids Research. 2024;52:D1180-D1192. doi:10.1093/nar/gkad1004.

[29] Lipinski CA, Lombardo F, Dominy BW, Feeney PJ. Experimental and computational approaches to estimate solubility and permeability in drug discovery and development settings. Advanced Drug Delivery Reviews. 2001;46:3-26. doi:10.1016/S0169-409X(00)00129-0.

[30] Kennard RW, Stone LA. Computer Aided Design of Experiments. Technometrics. 1969;11:137-148. doi:10.1080/00401706.1969.10490666.

[31] Bemis GW, Murcko MA. The properties of known drugs. 1. Molecular frameworks. Journal of Medicinal Chemistry. 1996;39:2887-2893. doi:10.1021/jm9602928.

[32] Sahigara F, Mansouri K, Ballabio D, Mauri A, Consonni V, Todeschini R. Comparison of Different Approaches to Define the Applicability Domain of QSAR Models. Molecules. 2012;17:4791-4810. doi:10.3390/molecules17054791.

[33] Gramatica P. Principles of QSAR models validation: internal and external. QSAR & Combinatorial Science. 2007;26:694-701. doi:10.1002/qsar.200610151.

[34] Rücker C, Rücker G, Meringer M. y-Randomization and Its Variants in QSPR/QSAR. Journal of Chemical Information and Modeling. 2007;47:2345-2357. doi:10.1021/ci700157b.
